# Nanoparticle delivery of miR-21-3p sensitizes melanoma to anti-PD-1 immunotherapy by promoting ferroptosis

**DOI:** 10.1136/jitc-2021-004381

**Published:** 2022-06-22

**Authors:** Weinan Guo, Zhenjie Wu, Jianru Chen, Sen Guo, Weiming You, Sijia Wang, Jinyuan Ma, Huina Wang, Xiangxu Wang, Hao Wang, Jingjing Ma, Yuqi Yang, Yangzi Tian, Qiong Shi, Tianwen Gao, Xiuli Yi, Chunying Li

**Affiliations:** 1 Department of Dermatology, Xijing Hospital, Fourth Military Medical University, Xi'an, Shaanxi, China; 2 Department of Bone and Soft Tissue Surgery, Guangxi Medical University Cancer Hospital, Nanning, Guangxi, China; 3 National & Local Joint Engineering Research Center of Biodiagnosis and Biotherapy, The Second Affiliated Hospital of Xi'an Jiaotong University, Xi'an, Shaanxi, China; 4 Department of Dermatology, Nanfang Hospital, Southern Medical University, Guangzhou, Guangdong, China; 5 Department of Oncology, Xijing Hospital, Fourth Military Medical University, Xi'an, Shaanxi, China

**Keywords:** drug therapy, combination, gene expression profiling, immunotherapy, melanoma, translational medical research

## Abstract

**Background:**

Although anti-programmed cell death protein 1 (PD-1) immunotherapy is greatly effective in melanoma treatment, low response rate and treatment resistance significantly hinder its efficacy. Tumor cell ferroptosis triggered by interferon (IFN)-γ that is derived from tumor-infiltrating CD8^+^ T cells greatly contributes to the effect of immunotherapy. However, the molecular mechanism underlying IFN-γ-mediated ferroptosis and related potentially promising therapeutic strategy warrant further clarification. MicroRNAs (miRNAs) participate in ferroptosis execution and can be delivered systemically by multiple carriers, which have manifested obvious therapeutic effects on cancer.

**Methods:**

MiRNAs expression profile in IFN-γ-driven ferroptosis was obtained by RNA sequencing. Biochemical assays were used to clarify the role of miR-21-3p in IFN-γ-driven ferroptosis and the underlying mechanism. MiR-21-3p-loaded gold nanoparticles were constructed and systemically applied to analyze the role of miR-21-3p in anti-PD-1 immunotherapy in preclinical transplanted tumor model.

**Results:**

MiRNAs expression profile of melanoma cells in IFN-γ-driven ferroptosis was first obtained. Then, upregulated miR-21-3p was proved to facilitate IFN-γ-mediated ferroptosis by potentiating lipid peroxidation. miR-21-3p increased the ferroptosis sensitivity by directly targeting thioredoxin reductase 1 (TXNRD1) to enhance lipid reactive oxygen species (ROS) generation. Furthermore, miR-21-3p overexpression in tumor synergized with anti-PD-1 antibody by promoting tumor cell ferroptosis. More importantly, miR-21-3p-loaded gold nanoparticles were constructed, and the systemic delivery of them increased the efficacy of anti-PD-1 antibody without prominent side effects in preclinical mice model. Ultimately, ATF3 was found to promote miR-21-3p transcription in IFN-γ-driven ferroptosis.

**Conclusions:**

MiR-21–3 p upregulation contributes to IFN-γ-driven ferroptosis and synergizes with anti-PD-1 antibody. Nanoparticle delivery of miR-21–3 p is a promising therapeutic approach to increase immunotherapy efficacy without obvious systemic side effects.

**Key messages**

- Tumor cell ferroptosis triggered by interferon (IFN)-γ derived from tumor-infiltrating CD8^+^ T cells greatly contributes to the effect of immunotherapy.
- MicroRNAs (miRNAs) are operative in ferroptosis execution and can be delivered systemically by multiple carriers in treating cancer; however, the role of miRNAs in anti-programmed cell death protein 1 (PD-1) immunotherapy-associated ferroptosis, as well as their therapeutic effect on melanoma, remains elusive.
- In the present study, through RNA sequencing and a panel of biochemical assays, we proved that miR-21-3p was a novel ferroptosis facilitator by targeting thioredoxin reductase 1 and could synergize with anti-PD-1 immunotherapy via the induction of tumor cell ferroptosis.
- The systemic delivery of miR-21-3p by gold nanoparticles robustly increased the efficacy of anti-PD-1 antibody without prominent side effects in preclinical mice model.
- This study demonstrates the great importance and translational potential of nanoparticle delivery of ferroptosis-associated miRNAs in melanoma immunotherapy, which warrants further investigations in future preclinical and clinical studies.

## Background

Originating from epidermal melanocytes, melanoma is the most malignant skin cancer. In the past few decades, the incidence of melanoma is gradually increasing and the survival of patients with advanced melanomas remains unoptimistic despite revolutionary progress in currently available targeted therapy and immunotherapy.^1 2^ Of the therapeutic options, neutralizing antibodies for blocking the interaction between programmed cell death protein 1 (PD-1) and PD-1 ligand (PD-L1) have been established to be greatly effective in treating melanoma.^2 3^ However, the low response rate and frequent occurrence of treatment resistance prominently hinder further improvement of patients’ outcomes.^4 5^ Therefore, it is necessary to investigate the mechanism underlying the resistance to anti-PD-1 immunotherapy and discover a novel combination therapeutic target to potentiate the treatment efficacy.

The induction of tumor cell death is the goal of all cancer treatments, including immunotherapy. Previous studies have demonstrated that tumor cell apoptosis triggered by cytotoxic factors like granzyme B, perforin and interferon-γ (IFN-γ) are mainly responsible for the cytotoxic effect of re-invigorated tumor-infiltrating CD8^+^ T cells after anti-PD-1 antibody treatment.^6 7^ Recently, several reports have pointed out that the activation of tumor-infiltrating CD8^+^ T cells induces ferroptosis of melanoma cells through the suppression of glutamate-cystine antiporter in an IFN-γ-dependent manner, highlighting the great contribution of ferroptosis, a novel cell death modality triggered by iron-dependent lipid peroxidation, to PD-1/PD-L1 blockade-based immunotherapy.^8–10^ More importantly, systemic application of agents like cyst(e)inase that facilitates tumor cell ferroptosis can significantly increase the efficacy of anti-PD-1 immunotherapy in transplanted tumor model,^9 11^ which indicates the great translational potential of potentiating ferroptosis in melanoma immunotherapy. Of note, upon the treatment with ferroptosis inducers, some crucial mediators are simultaneously regulated to promote or suppress the execution of ferroptosis. For example, the activation of ATF4 promotes the expression of unfolded protein response regulator HSPA5 to suppress GPX4 degradation and therefore inhibit ferroptosis.^12^ On the other hand, the upregulation of ATF3 potentiates ferroptosis via the transcriptional suppression of system Xc^−^ expression.^13^ Hence, to further elucidate the upstream regulators of ferroptosis might help to provide novel therapeutic targets for amplifying the efficacy of anti-PD-1 immunotherapy.

MicroRNAs (miRNAs) are a class of endogenous small non-coding RNAs with 19–25 nucleotides that regulate gene expression mostly through the interaction with the 3’ untranslated region (3’UTR) of target mRNA.^14^ MiRNAs are operative in ferroptosis execution.^15 16^ Specifically, miR-137 exerts its inhibitory effect on ferroptosis via the suppression of glutamine transporter SLC1A5 in melanoma cells.^17^ In addition, miR-522 secreted by cancer-associated fibroblasts suppresses tumor cell ferroptosis to mediate acquired chemo-resistance in gastric cancer.^18^ These reports demonstrate the critical regulatory effect of miRNAs on ferroptosis and their potential as therapeutic targets. Intriguingly, some miRNAs delivered systemically by nanoparticle or alternative carriers exhibit obvious treatment effects on cancer with relatively high specificity in targeting tumor cells.^19–22^ Moreover, some clinical trials reveal that miRNAs-based mimic drugs have potent antitumor capacity and a subset of patients can gain encouraging responses (NCT04675996 and NCT02369198).^23–26^ Therefore, the employment of nanoparticle-based delivery of candidate miRNAs in the regulation of ferroptosis might be a promising synergistic approach for cancer immunotherapy, but the roles of miRNAs in anti-PD-1 immunotherapy-associated ferroptosis, as well as their therapeutic effect on melanoma, should be thoroughly investigated.

In the present study, we first investigated the miRNAs expression profile of melanoma cells in response to IFN-γ-potentiated ferroptosis that mimicked the effect of activated tumor-infiltrating CD8^+^ T cells in immunotherapy. Then, miR-21–3 p was identified as one of the most significantly upregulated miRNAs in the expression profile analysis, of which the role in regulating ferroptosis and the underlying mechanism were demonstrated. Subsequently, overexpression of miR-21–3p in tumor *ex vivo* and systemic delivery of miR-21–3p-loaded gold nanoparticle *in vivo* respectively testified the synergized therapeutic effect on melanoma along with anti-PD-1 antibody. Ultimately, the upstream regulator responsible for the increase of miR-21–3p in ferroptosis was also investigated.

## Methods

### Cell culture and reagents

Human melanoma cell lines WM793B, A2058, A375, Hs294T and mouse melanoma cell line B16F10 were purchased from the American Type Culture Collection. A2058, A375, Hs294T and B16F10 cells were cultured in Dulbecco’s Modified Eagle Medium (Thermo Fisher Scientific, Waltham, Massachusetts, USA) supplemented with 10% fetal bovine serum (Invitrogen, Carlsbad, California, USA) and 1% penicillin-streptomycin (Invitrogen). WM793B cell line was maintained in MCDB153 medium (Sigma-Aldrich) with 2% fetal bovine serum (Invitrogen). Human melanoma cell lines UACC62 and UACC257 were given from Dr. Schrama in University Hospital Würzburg in Germany in 2016, and were cultured in RPMI 1640 medium (Hyclone) supplemented with 10% fetal bovine serum (Invitrogen). All these melanoma cell lines were authenticated by short-tandem repeat fingerprinting by Fourth Military Medical University in 2016, and these cell lines show no mycoplasma contamination. IFN-γ was purchased from R&D Systems (Minneapolis, Minnesota, USA). Liproxstatin-1 (HY-12726), (1S,3R)-RSL3 (HY-100218A), erastin (HY-15763), ferrostatin-1 (HY-100579) were purchased from MedChemExpress (Monmouth Junction, New Jersey, USA). agomiR-21–3p (hsa-miR-21–3p) and antagomiR-21–3p (hsa-miR-21–3p antagomiR) were purchased from RiboBio (Guangzhou, China).

### Clinical specimens

Tissue samples for immunohistochemical and quantitative real-time (qRT)-PCR analysis were taken from 31 patients with melanoma after the histological confirmation. All the clinical specimens were obtained in Department of Dermatology, Xijing Hospital, the Fourth Military Medical University.

### MicroRNA isolation, complementary DNA synthesis and real-time qRT-PCR

MiRNA isolation, complementary DNA (cDNA) synthesis and qRT-PCR were performed using kits according to the manufacturer’s instruction as described before.^27^ Total RNA was extracted using TRIzol reagent (cat. 15596018, Invitrogen). cDNA was synthesized from miRNA using miRNA cDNA First Strand Synthesis kit (cat. KR211-01, Tiangen Biotech, Beijing, China) and qRT-PCR was performed using miRNA SYBR qRT-PCR Kit (cat. FP411-01, Tiangen Biotech). The primers of hsa-miR-21–3p (cat. CD201-0093), has-miR-22–3p (cat. CD201-0305), has-miR-210–3p (cat. CD201-0293), has-miR-7–5p (cat. CD201-0141), has-miR-10a-5p (cat. CD201-0515), has-miR-9–5p (cat. CD201-0142) and hsa-U6 (cat. CD201-0145) were purchased from Tiangen Biotech. The primer of mmu-miR-21–3p (cat. MIRAP01226) was purchased from Sigma-Aldrich, and mmu-U6 was purchased from Genepharma. Threshold cycle (CT) for each miRNA was determined using the iQ5-standard Edition Optical System V.2.1 (Bio-Rad, Hercules, California, USA). Relative quantification was performed according to the ΔΔCT method, and results were expressed in the linear form using the formula 2-^ΔΔCT^. U6 miRNA was used as an internal control.

### Luciferase reporter assay

Transfections of melanoma cells were performed using Lipofectamine 3000 (cat. L3000015, Invitrogen) according to the manufacturer’s instructions as described before.^27^ In brief, luciferase reporter vectors (100 ng) (thioredoxin reductase 1 (TXNRD1) wild-type (WT), TXNRD1 MUT and the empty vectors, GenePharma, Shanghai, China) were individually transfected into melanoma cells along with 100 nM agomiR-21–3 p (RiboBio) or 100 nM agomiR-negative control (RiboBio). Forty-eight hours after transfection, luciferase assays were performed using the Dual-Luciferase Reporter Assay System (Promega, Madison, Wisconsin, USA) according previously described.^27^ At least three independent experiments were performed and transfection efficiency was normalized using Renilla luciferase.

### Immunofluorescence staining analysis

Paraffin-embedded tissue sections were deparaffinized and rehydrated with graded ethanol dilutions. After antigen retrieval in Tris-EDTA buffer (10 mM, pH 9.0), the paraffin tissue sections were blocked with goat serum for 30 min. And then immunofluorescence staining was performed by incubating the paraffin tissue sections with a primary antibody (cat. ab179800, rabbit monoclonal to anti-COX2 (PTGS2), 1:100, Abcam, Cambridge, Massachusetts, USA; cat. 67728-1-Ig, mouse monoclonal anti-TXNRD1 antibody, 1:100, Proteintech, Rosemont, USA; cat. bs-0480R, rabbit anti-IFN-γ antibody, 1:500, Biosynthesis Biotechnology, Beijing, China; cat. MA1-145, rat monoclonal anti-CD8 antibody, 1:200, Invitrogen; cat. ab254268, rabbit monoclonal anti-ATF3 antibody, 1:100, Abcam) overnight at 4°C, followed by 1 hour incubation with appropriate secondary antibodies (cat. EK022, goat antirabbit IgG H&L (Cy3), 1:100; cat. EK041, goat antirat IgG H&L (FITC), 1:100 and cat. EK013, goat antimouse IgG H&L (FITC), 1:100, Zhuangzhibio, Xi’an, China). Hoechst 33258 (cat. C1011, Beyotime, Shanghai, China) was used as a counterstain. Tissue sections were analyzed by confocal laser scanning microscopy (FV-1000, Olympus, Tokyo, Japan).

### RNA sequencing

A375 melanoma cells were treated with either RSL3 or RSL3 combined with IFN-γ, respectively. Total RNA was extracted using TRIzol reagent (Invitrogen) following the manufacturer’s instructions. A total amount of 3 μg of RNA per sample was used as input material for the RNA sample preparations. Sequencing libraries were generated using NEBNext Ultra Directional RNA Library Prep Kit for Illumina (NEB, USA) following the manufacturer’s recommendations. After cluster generation, the library preparations were sequenced on an Illumina Hiseq 2500 platform by CapitalBio Technology as previously described.^28^ Sequencing data are accessible through GEO series accession numbers GSE186497.

### Immunohistochemical staining analysis

Paraffin-embedded heart, kidney, spleen, lung and liver tissues of mice were deparaffinized and rehydrated with graded ethanol dilutions. After antigen retrieval in Tris-EDTA Buffer (10 mM Tris Base, 1 mM EDTA solution, 0.05% Tween-20, pH 9.0), goat serum was added to block non-specific binding for 30 min. Tissue section was incubated in Fast Red solution, and subsequently counterstained with hematoxylin and mounted with glycerol.

For the analysis of CD8α in paraffin-embedded melanoma tissues from patients and ATF3 and PTGS2 in tumor tissue microarray (TMA), tissue sections were de-paraffinized and rehydrated with graded ethanol dilutions. After antigen retrieval in Tris-EDTA buffer (10 mM Tris Base, 1 mM EDTA solution, 0.05% Tween-20, pH 9.0), goat serum was added to block non-specific binding for 30 min. Tissue sections were then incubated with CD8 antibody (cat. ZA-0508, rabbit monoclonal antibody, 1:1, ZSGB-BIO, China), or a primary antibody (cat. ab254268, rabbit monoclonal anti-ATF3 antibody, 1:200, Abcam; cat. ab179800, rabbit monoclonal to anti-COX2 (PTGS2), 1:100, Abcam) at 4°C overnight, followed by antirabbit alkaline phosphatase secondary antibody (cat. cw2069s, 1:1, Cwbio, China). The section was then incubated in Fast Red solution, and subsequently counterstained with hematoxylin and mounted with glycerol. The evaluation of staining scores was described previously.^29^ Briefly, the percentages of staining-positive cells were scored into four categories: 0 (0%), 1 (1%–33%), 2 (34%–66%) and 3 (67%–100%). The staining intensities were scored into four grades: 0 (none), 1 (weak), 2 (moderate) and 3 (strong). The final staining score was defined as the product of the percentage and the intensity scores.

### Fabrication of miR-21-3p-AuNp

The solution of tetrachloroauric acid (HAuCl_4_·XH_2_O, 1 mL, 10 mM) was mixed with 9 mL 4-(2-hydroxyethyl)-1-piperazineethanesulfonic acid (HEPES) buffer (pH 7.4, 50 mM). After 10 min magnetic stirring, the solution color changed from golden yellow to wine red, which is the Au-core. Meanwhile, 1 mL tetrachloroauric acid (10 mM) solution was added into 9 mL buffer (50 mM HEPES, 20% ethyl alcohol) which has dissolved 1 OD microRNA (miR-21–3 p-SH) and 3 mg SH-PEGn-NH_2_ (MW 2000 Da). Then it was mixed with the prepared Au-core solution (10 mL) under magnetic stirring. Finally, excess reactants were removed by dialysis tubing (cut-off, 10 kDa) and washed twice by distilled water. Finally, we obtained miR-21–3 p-AuNp.

### Physicochemical properties of miR-21-3p-AuNp and its intergradations

The morphology and lattice structure were observed on transmission electron microscopy (TEM), which was performed on an HT7700 operated at an acceleration voltage of 100 kV. One portion of the pellet was placed onto a carbon-coated copper grid for imaging with high-resolution TEM and selected area electron diffraction (JEM-200CX, Horiba, Japan). Energy-dispersive spectroscopy analysis was performed on the nanoparticles formed from Au^3+^ at 20 kV accelerating voltage and 133 eV resolution on a scanning area of 1×1 µm using an EX-250 spectrometer (Horiba). The hydrodynamic size distribution (1 mg/mL in phosphate-buffered saline (PBS), 1 mL) was obtained from the dynamic light scattering (DLS) measurement (Malvern Zetasizer Nano ZS system). For zeta potential measurement, the nanoparticles (1 mg/mL, 1 mL) were incubated with PBS at different pH at 37°C for 30 min, and measured by DLS. The surface chemical structure of modified nanocrystals was evaluated by Fourier transform infrared spectroscopy (Nicolet 6700) and UV-Vis absorption spectra (Shimadzu 3000 spectrophotometer).

### Animal experiments

**Figure 1:**
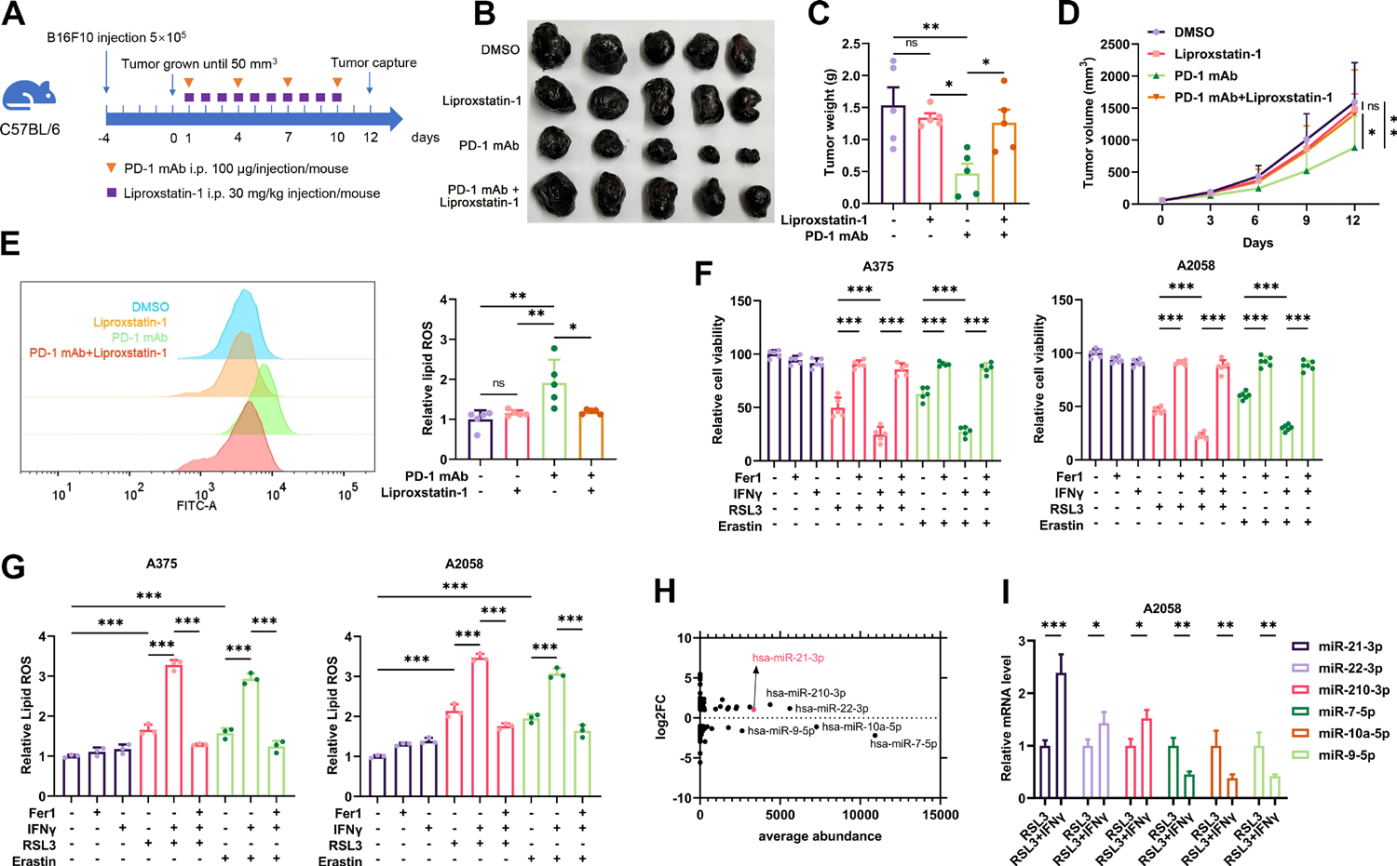
The expression profile of miRNAs in IFN-γ-potentiated ferroptosis in melanoma. (A) A schematic view of the treatment plan that C57BL/6 mice burdened with B16F10 tumors received anti-PD-1 antibody and liprostatin-1 treatment as indicated. (B-D) Images of isolated tumors from mice that received indicated treatment. Tumor volumes and weights in each group were calculated and displayed in (C) and (D). (E) Relative lipid ROS in isolated transplanted tumors with indicated treatment. (F-G) Relative cell viability and lipid ROS level in A2058 and A375 melanoma cells after indicated treatment with IFN-γ, RSL3, erastin and Fer-1. (H) The average abundance of the differentially expressed miRNAs related to (online supplemental figure S1D). (I) The relative expression of the most significantly differentially-expressed miRNAs after the treatment with RSL3 or RSL3 combined with IFN-γ in A375 melanoma cell. Erastin was used at 10 µM in both cell lines. RSL3 was used at 0.5 µM in A375 and 1 µM in A2058 cell line. Fer-1 was used at 2 µM in both cell lines. IFN-γ was used at 50 ng/mL in both cell lines. Data represent the mean±SD of triplicates. P value was calculated by two-tailed Student’s t-test. ^*^P<0.05, ^**^p<0.01, ^***^p<0.001. IFN, interferon; miRNA, microRNA; ns, non-significant; PD-1, programmed cell death protein 1; ROS, reactive oxygen species.

In liproxstatin-1 rescue experiment, 5×10^5^ B16F10 cells were subcutaneously injected into the right flank of C57BL/6 mice. When the tumor grows to 50 mm^3^, 100 µg anti-PD-1 antibody (Bio X Cell, USA), 30 mg/kg liproxstatin-1 (MedChemExpress, USA) or both were administered intraperitoneally to each mouse. Anti-PD-1 antibody was administered every 3 days and liproxstatin-1 was administered every day. Tumor diameters were measured using calipers. After around 16 days after the transplantation as indicated in figure 1A, the mice were sacrificed and the tumors were harvested and photographed. After the examination of their weights, tumors were fixed in 4% paraformaldehyde overnight. Paraffin-embedded sections were processed to immunofluorescence staining analysis of T cell infiltrate and ferroptosis-marker expression.

10.1136/jitc-2021-004381.supp1 Supplementary data

To determine whether tumorous miR-21–3p upregulation was capable of contributing to anti-PD-1 immunotherapy via the regulation of tumor cell ferroptosis in melanoma, 5×10^5^ B16F10 cells pretransfected with vector encoding miR-21–3p or miR-NC were subcutaneously injected into the right flank of C57BL/6 mice, which then received the systemic administration of anti-PD-1 antibody and liproxstatin-1 as indicated in figure 4A. Tumor diameters were measured using calipers. To obtain the stable overexpression of miR-21–3p in B16F10 melanoma cells, the plasmid vectors encoding miR-21–3p were used to transfect cells according to the manufacturer’s recommended procedures of Lipofectamine 3000 (Invitrogen). The plasmid vectors were purchased from Genechem, China. After around 16 days after the transplantation as indicated in figure 4A, the mice were sacrificed and the tumors were harvested and photographed. After the examination of their weights, tumors were fixed in 4% paraformaldehyde overnight. Paraffin-embedded sections were processed to immunofluorescence staining analysis. The comparisons of tumor volumes and tumor weights between different groups were analyzed by two-tailed Student’s unpaired t-test.

In systemic delivery of miR-21–3p by gold nanoparticles (AuNp) experiment, 5×10^5^ B16F10 cells were subcutaneously injected into the right flank of C57BL/6 mice. When the tumor grows to 50 mm^3^, 100 µg anti-PD-1 (Bio X Cell, USA), 10 mg/kg AuNp or miR-21–3p-AuNp, 30 mg/kg liproxstatin-1 were administered intraperitoneally to each mouse. Anti-PD-1 (Bio X Cell) and miR-21–3p-AuNp were administered every 3 days and liproxstatin-1 was administered every day. After around 16 days after the transplantation as indicated in online supplemental figure 3B, the mice were sacrificed and the tumors were harvested and photographed. After the examination of their weights, tumors were fixed in 4% paraformaldehyde overnight. Paraffin-embedded sections were processed to immunofluorescence staining analysis. The comparisons of tumor volumes and tumor weights between different groups were analyzed by two-tailed Student’s unpaired t-test.

For T-cell depletion studies, antibodies to deplete CD8 (clone 2.43; Bio X Cell) were injected intraperitoneally at 100 μg per mouse on days −7 to –5, −2, +1, +4 and every other 3 days afterwards until completion of the study as displayed in online supplemental figure 4E.

To examine the intratumor levels of IFN-γ, tumor necrosis factor (TNF)-α, interleukin (IL)-6, IL-10, C-X-C motif chemokine ligand (CXCL)9 and CXCL10, single cell suspension of B16F10 transplanted tumors were obtained by rapid and gentle stripping, physical grinding in cold PBS and filter filtration (FALCON cell strainer, 352350, Corning, New York, USA). These cytokines and chemokines were detected with ELISA kits (all from Elabscience) according to the manufacturer’s instructions.

### Flow cytometry analysis of immune cells in TME

All flow cytometry antibodies and agents for analyzing immune cells in TME were purchased from BioLegend, San Diego, California, USA. Single cell suspension of B16F10 transplanted tumors were obtained by rapid and gentle stripping, physical grinding in cold PBS and filter filtration (FALCON cell strainer, 352350, Corning). After getting rid of dead cells with Zombie UV Fixable Viability Kit (423108), cells were stained with Pacific Blue-CD45 (cat. 157212), APC-CD3 (cat. 100236), PE-CD4 (cat. 116006), PECY7-CD8a (cat. 100722), PE-CD11b (cat. 101208) and APC-F4/80 (cat. 123116) for 30 min. After fixation and permeabilization by True-Nuclear Transcription Factor Buffer Set (cat. 424401), intracellular GZMB and IFN-γ were stained using PE-GZMB (cat. 372208) and APC-IFN-γ (cat. 505810) antibody. Stained cells were analyzed by BD LSRFortessa. Data were further analyzed by FlowJo V.10.0 software.

### Toxicity studies

To determine potential toxicities of miR-21–3p-AuNp including the nephrotoxicity, we monitored body weights of all mice over the course of treatment and measured hematological indexes as well as organ function indexes after 13 days of treatment. Control mice were implanted with tumor, and only received the treatment of dimethyl sulfoxide. Forty-eight hours after the final infusion, mice were anesthetized and blood was collected for complete blood count determinations, including a white blood cell count with differential, a red blood cell count, hemoglobin and a platelet count. Besides, blood serum was collected, and cytokines IL-2, IFN-γ, TNF-α, IL-6 and eosinophil and erythropoietin were measured by using quantitative ELISA kits according to the manufacturer’s instructions. Animals were then euthanized with carbon dioxide to retrieve organs, which were washed with deionized water before fixation in 4% paraformaldehyde. The tissues were processed routinely, and sections were stained with H&E.

### Signature score computation

A gene set including *CD8A*, *GZMA*, *GZMB*, *IFNG*, *CXCL9*, *CXCL10*, *PRF1* and *TBX21* was used for CD8^+^ T effector signature score, and a gene set that is upregulated by erastin treatment and reversed by co-treatment with β-mercaptoethanol in HT-1080 cells was used for ferroptosis response signature.^30 31^ The signature score was calculated via gene set variation analysis (GSVA) and assumed to be normal random variable (~N(0, 1)) as previously described.^32^ In addition, the IFN-γ enrichment scores of melanoma cell lines were also calculated by GSVA based on the expression of the gene set including *IFNGR*1, *IFNGR2*, *JAK1*, *JAK2*, *STAT1* and *IRF1*.

### Statistical analysis

Each experiment was performed at least for three times, and statistical analyses of the data were performed using unpaired, two-tailed Student’s t-tests built into GraphPad Prism (GraphPad Software V.3.0; San Diego, California, USA). One-way analysis of variance was employed to compare the differences among multiple groups. Survival functions were estimated by Kaplan-Meier methods and log-rank test was used to calculate statistical differences. Pearson’s correlation was used to evaluate the association between the expressions of two genes. All the data are expressed as the mean±SD. P values <0.05 were considered statistically significant.

## Results

### The expression profile of miRNAs in IFN-γ-driven ferroptosis in melanoma

Previous studies have reported that anti-PD-1 immunotherapy could re-invigorate the effector function of CD8^+^ T cells in TME, which triggered tumor cell ferroptosis by secreting IFN-γ and suppressing the expression of subunits of the glutamate-cystine antiporter system Xc^−^.^8 9^ To confirm whether tumor cell ferroptosis is responsible for the therapeutic effect of anti-PD-1 antibody treatment, we employed liproxstatin-1, a ferroptosis-specific inhibitor *in vivo*,^33^ to treat C57BL/6 mice implanted with B16F10 melanomas and receiving anti-PD-1 antibody treatment (figure 1A). As a result, while anti-PD-1 antibody alone led to the reduction of tumor growth, the systemic administration of liproxstatin-1 could prominently suppress melanoma regression resulting from immunotherapy (figure 1B–D). Immunofluorescence staining analysis consistently revealed that anti-PD-1 antibody induced the expression of ferroptosis marker prostaglandin-endoperoxide synthase 2 (PTGS2) in the tumor, but co-administration with liproxstatin-1 could significantly reverse this alteration (online supplemental figure S1A). In parallel, flow cytometry analysis detecting intratumoral lipid ROS level showed that liproxstatin-1 treatment restrained the accumulation of lipid ROS induced by anti-PD-1 antibody (figure 1E). IFN-γ is the main effector that triggers tumor cell ferroptosis in immunotherapy,^9^ for this assessment, we treated human melanoma cell lines A375 and A2058 with IFN-γ and ferroptosis inducer erastin or RSL3 to mimic immunotherapy-induced tumor cell ferroptosis *in vitro* as previously described.^9^ As expected, IFN-γ treatment enhanced erastin-induced or RSL3-induced melanoma cell ferroptosis with concomitant increased generation of lipid peroxidation end product malondialdehyde (MDA) and lipid ROS, which was blocked after the further treatment with ferroptosis-specific inhibitor ferrostatin-1 (figure 1F,G, online supplemental figure S1B, C). Therefore, the blockade of ferroptosis was capable of abolishing the efficacy of anti-PD-1 immunotherapy in melanoma.

According to previous studies, some intrinsic signaling pathways would be activated or suppressed on the treatment with ferroptosis inducer to mediate the execution of cell death.^34^ The essential role of ferroptosis in contributing to tumor immunotherapy necessitates counteracting the protective signaling (or promoting the facilitative signaling) in augmenting immunotherapy efficacy. Accumulative evidence has revealed that the dysregulation of miRNAs was highly involved in modulating ferroptosis.^15^ However, the role of miRNAs in IFN-γ-driven ferroptosis and related immunotherapy has not been thoroughly investigated. To this end, we employed RNA sequencing to obtain the expression profile of miRNAs in A375 melanoma cells treated with either RSL3 or RSL3 combined with IFN-γ, respectively. Compared with RSL3 monotreatment group, there were 86 miRNAs displaying >2-fold change and 70 miRNAs displaying <0.5-fold change in the RSL3 combined with IFN-γ treatment group, delineating the specific expression profile of miRNAs in IFN-γ-driven ferroptosis in melanoma cell (online supplemental figure S1D, online supplemental table S1). In particular, several differentially-expressed miRNAs were of relative higher abundance than other miRNAs that manifested more significant alteration after the co-treatment with RSL3 and IFN-γ (figure 1H, online supplemental table S1). Our subsequent qRT-PCR assay verified the alterations of these candidate miRNAs including miR-21-3p, miR-22-3p, miR-210-3p, miR-7-5p, miR-10a-5p and miR-9-5p after indicated stimulations, which displayed the same trend as the results of RNA sequencing (figure 1I). Of note, the expression level of miR-21–3p was markedly higher than that of miR-22–3p and miR-210-3p after the combined treatment with RSL3/erastin and IFN-γ compared with RSL3/erastin monotreatment (online supplemental figure S1E). In addition, these candidate miRNAs were also robustly induced after RSL3 or erastin monostimulation compared with the control group, with the level of miR-21–3p revealing more significant upregulation (online supplemental figure S1E). Taken together, these miRNAs might be ferroptosis-responsive and the level of which could be further elevated after the co-treatment with IFN-γ in melanoma.

10.1136/jitc-2021-004381.supp2 Supplementary data

### MiR-21-3p upregulation contributes to ferroptosis via the potentiation of lipid peroxidation

**Figure 2:**
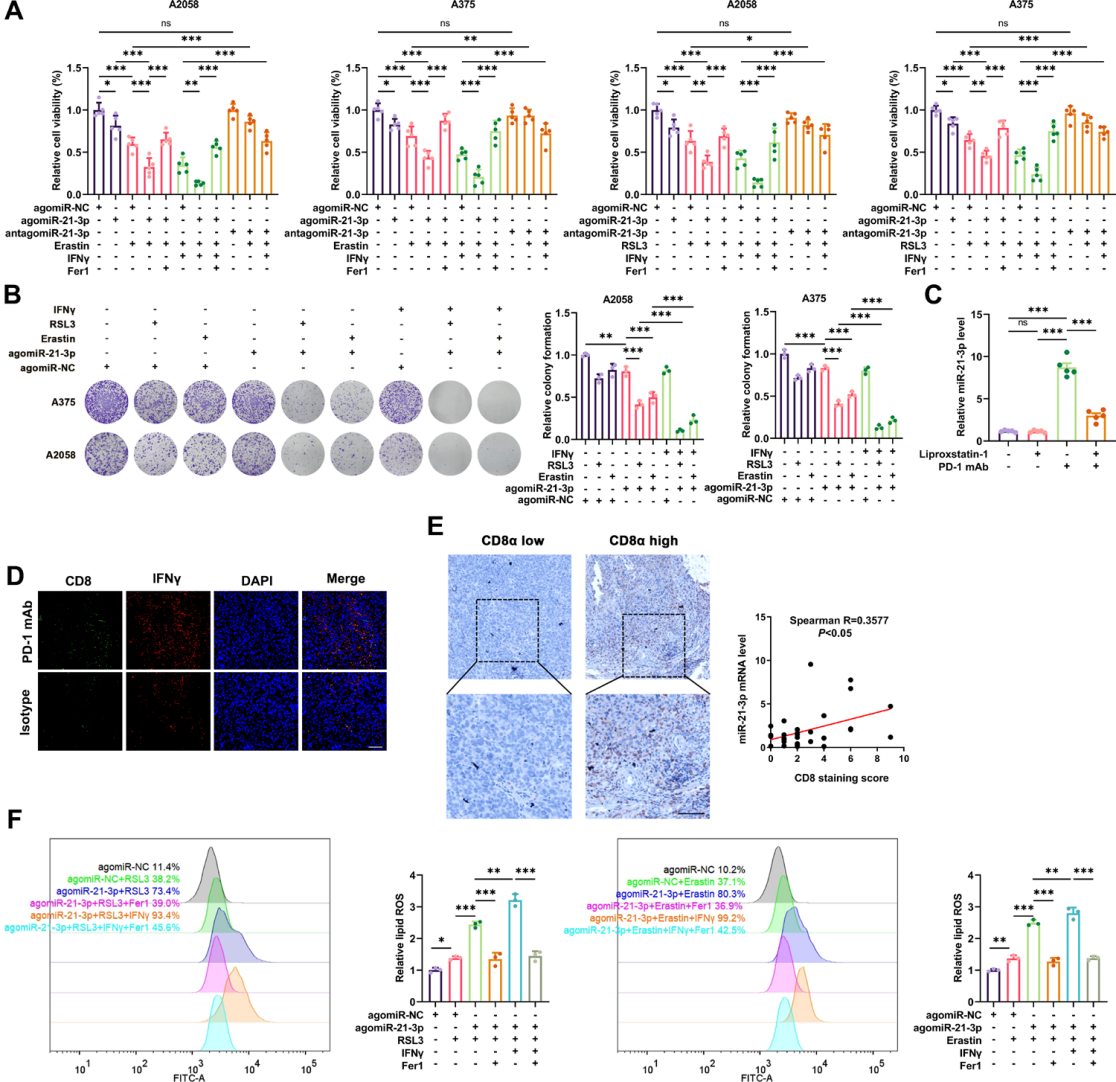
MiR-21–3p upregulation contributes to IFN-γ-mediated ferroptosis in melanoma by promoting lipid peroxidation. (A-B) Relative cell viability and colony formation after the intervention of miR-21–3p in melanoma cells treated with both ferroptosis inducers and IFN-γ. (C) The level of miR-21–3p detected by quantitative real-time-PCR in isolated transplanted tumors from mice that received indicated treatment related to figure 1A. (D) Immunofluorescence staining of CD8 and IFN-γ in isolated tumors from mice that received anti-PD-1 antibody. Scale bar=50 µm. (E) The correlation between miR-21-3p level and CD8 staining score in a cohort of 31 melanoma tissues. (F) Relative lipid ROS level in ferroptosis inducer-treated melanoma cells with the intervention of miR-21–3p. AgomiR-NC, agomiR-21–3p and antagomiR-21–3p were all used at 100 nM in both cell lines. Erastin was used at 10 µM in both cell lines. RSL3 was used at 0.5 µM in A375 and 1 µM in A2058 cell line. Fer-1 was used at 2 µM in both cell lines. IFN-γ was used at 50 ng/mL in both cell lines. Data represent the mean±SD of triplicates. P value was calculated by two-tailed Student’s t-test. ^*^P<0.05, ^**^p<0.01, ^***^p<0.001. IFN, interferon; ns, non-significant; PD-1, programmed cell death protein 1; ROS, receiver operating characteristic.

Considering miR-21–3p was increased more prominently after the combined treatment with erastin/RSL3 and IFN-γ and was of relatively higher abundance among the upregulated miRNAs, we went on to investigate the role of miR-21–3 p in ferroptosis. To this end, we treated A2058 and A375 cells with chemically engineered agomiRs and antagomiRs that could specifically potentiate or suppress the function of miR-21–3p. As cell counting kit-8 (CCK8) assay revealed, agomiR-21–3 p facilitated the cell death induced by erastin/RSL3 monotreatment or with combined IFN-γ treatment, and this effect could be mostly blocked by ferrostatin-1 (figure 2A). In contrast, antagomiR-21–3p could effectively antagonize the lethal effect of ferroptosis inducers (figure 2A). Concurrent colony formation assay also revealed that agomiR-21–3p facilitated ferroptosis induced by RSL3/erastin monotreatment, which could be forwardly enhanced by the combined treatment with IFN-γ in both A2058 and A375 cell lines (figure 2B). These results suggested that the upregulation of miR-21–3p was greatly implicated in IFN-γ-driven ferroptosis in melanoma. Furthermore, the transwell assay showed that agomiR-21–3p treatment had little impact on the invasion and migration of both A2058 and A375 melanoma cells (online supplemental figure S2A), suggesting that miR-21–3p might be dispensable for the invasion and metastasis of melanoma.

To further confirm the upregulation of miR-21–3p in IFN-γ-stimulated ferroptosis and the potential clinical implication in anti-PD-1 immunotherapy, we employed qRT-PCR analysis to detect the expression of miR-21–3p *in vivo*. We obtained the transplanted tumors isolated from C57BL/6 mice implanted with B16F10 melanoma cells and receiving anti-PD-1 immunotherapy. The level of miR-21–3p in the tumor was prominently increased after anti-PD-1 antibody treatment (figure 2C), in keeping with the increase of IFN-γ in tumor-infiltrating CD8^+^ T cells (figure 2D). Moreover, the level of intratumoral miR-21–3p was significantly reduced after the co-administration with liproxstatin-1 (figure 2C). The qRT-PCR and immunohistochemical staining assays in a cohort of 31 melanoma tissues revealed the positive correlation between miR-21–3p levels and the staining scores of CD8 (figure 2E), proving the close relationship between miR-21–3p and CD8^+^ T cell-dependent antitumor immunity. We also examined the level of miR-21–3p in human melanoma cell lines (A375 and A2058), fibroblasts (human dermal fibroblast-adult cell line),^35^ keratinocytes (HaCat cell line) and peripheral blood mononuclear cells, all of which are critical components in TME of melanoma. The relative level of miR-21–3p is higher in melanoma cells compared with other types of cells (online supplemental figure S2B). Since that the quantity of melanoma cells dominates within TME compared with immune cells, fibroblasts and other types of cells, it is of high possibility that miR-21–3p level in melanoma tissues is largely from melanoma cells.

Thereafter, to further explore the mechanism underlying the role of miR-21–3p in ferroptosis, we analyzed lipid peroxidation and iron accumulation which are two important signaling events in triggering ferroptosis.^36^ Concerning MDA was among the most important end-products of lipid peroxidation, we testified whether miR-21–3p regulated MDA content in melanoma cell ferroptosis. As a result, the potentiation of MDA induced by either erastin or RSL3 monotreatment could be further intensified after the overexpression of miR-21–3p in melanoma cells. Besides, agomiR-21–3p further promoted the generation of MDA resulting from the combined treatment with RSL3/erastin and IFN-γ in both A2058 and A375 cell lines (online supplemental figure S2C). In contrast, antagomiR-21–3p treatment prominently ameliorated MDA generation in melanoma cells after the stimulation with erastin/RSL3 or the combined treatment with IFN-γ (online supplemental figure S2C). Consistently, lipid ROS generation assayed by flow cytometry using the fluorescent probes C11-BODIPY unveiled that the agomiR-21–3p potentiated lipid peroxidation induced by erastin/RSL3 monotreatment or the combined treatment with IFN-γ (figure 2F).

The overload of intracellular ferrous iron (Fe^2+^) is also a crucial event for triggering ferroptosis according to previous reports.^37^ Treatment with iron chelators like deferoxamine could prevent multiple cells from undergoing ferroptosis.^38^ As expected, the intracellular level of Fe^2+^ was significantly increased after the stimulation with erastin/RSL3 compared with the control group, and was further potentiated after the combined treatment with IFN-γ (online supplemental figure S2D). However, agomiR-21–3p had little impact on intracellular Fe^2+^ level under the treatment with erastin/RSL3 or the combined treatment with IFN-γ (online supplemental figure S2D). Taken together, the above results demonstrated that upregulated miR-21–3p level contributed to IFN-γ-stimulated ferroptosis mainly by promoting the generation of lipid peroxidation, rather than affecting intracellular Fe^2+^ level in melanoma.

We have also emphasized on considering the involvement of IFN-γ pathway variations in different melanoma cells as previously described.^39^ To this end, we turned to CCLE database^40^ and found that there were transcriptional data of six melanoma cell lines that are also currently available in our laboratory (A2058, UACC62, UACC257, A375, Hs294T and WM793B) (online supplemental table S2). While there were no any mutations or genomic variations in *IFNGR1*, *IFNGR2*, *JAK1*, *JAK2*, *STAT1* and *IRF1* in IFN-γ pathway (data not shown), the transcriptional expressions of these molecules in different cell lines display remarkable heterogeneity. GSVA of these molecules has been employed to calculate the enrichment score to reveal the activation status of IFN-γ pathway in theses cell lines. As was shown, the GSVA enrichment score of IFN-γ pathway was highest in WM793B cell line, whereas A2058 and A375 cell lines harbored relatively low endogenous IFN-γ enrichment score (online supplemental figure S2E). This result indicates that WM793B might be more responsive to IFN-γ stimulation and its mediated ferroptosis. We treated A2058, A375 and WM793B cell lines with the same concentration of IFN-γ, and the expressions of IRF1 and phosphor-STAT1 were increased more prominently in WM793B cell line compared with the other two (online supplemental figure S2F). Intriguingly, the combination of IFN-γ and RSL3/erastin could induce more prominent tumor cell ferroptosis in WM793B cell line (online supplemental figure S2G-H, figure 2A). Besides, agomiR-21–3p treatment could promote more significant tumor cell ferroptosis induced by RSL3/erastin or RSL3/erastin combined with IFN-γ in WM793B cell line compared with the other two cell lines (online supplemental figure S2G–H, figure 2A). In line with this, lipid ROS generation displayed more significant alteration in WM793B cell line compared with A2058 cell line in corresponding group with identical treatments (online supplemental figure S2I). Therefore, the heterogeneity of IFN-γ pathway in different melanoma cells is decisive for the response to IFN-γ-driven ferroptosis. IFN-γ pathway deficiency might impair the induction of ferroptosis driven by IFN-γ and agomiR-21–3p, thus leading to deficient antitumor immunity.

10.1136/jitc-2021-004381.supp3 Supplementary data

### MiR-21-3p directly targets TXNRD1 to facilitate ferroptosis

**Figure 3:**
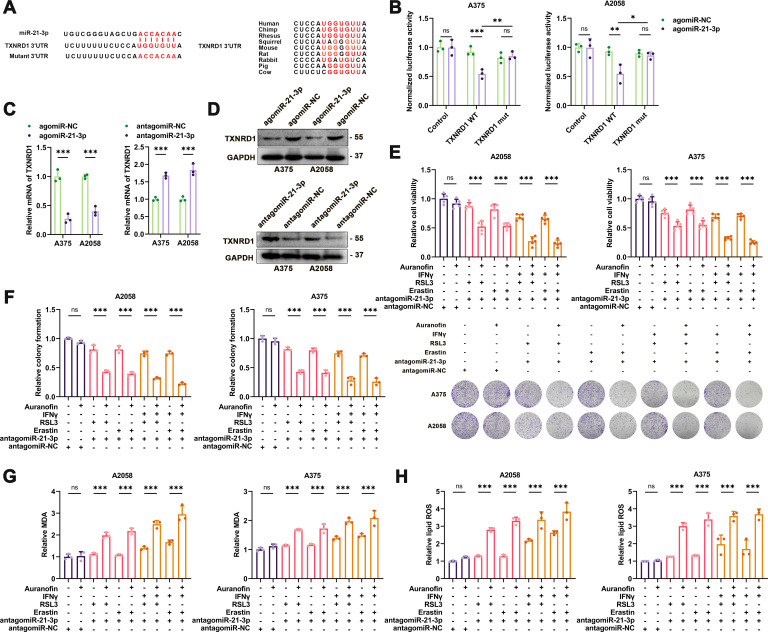
MiR-21–3p directly targets TXNRD1 to promote ferroptosis. (A) Schematic illustration of the sequence of hsa-miR-21–3p and its complementary sequence in 3’UTR of TXNRD1 mRNA in distinct species. (B) Luciferase activity assays using luciferase reporters with wild-type TXNRD1 3’UTR or mutant TXNRD1 3’UTR were performed with co-transfection of agomiR-21–3p or negative control into A375 and A2058 melanoma cells. (C) Relative mRNA level of TXNRD1 in melanomas with the intervention of miR-21–3p. (D) Immunoblotting analysis of TXNRD1 in melanomas with the intervention of miR-21–3p. (E-F) Relative cell viability and colony formation of ferroptosis inducer-treated melanoma cells with both the intervention of miR-21–3p and pharmacological inhibition of TXNRD1 by Auranofin. (G-H) Relative intracellular MDA content and lipid ROS level in ferroptosis inducer-treated melanoma cells with both the intervention of miR-21–3p and pharmacological inhibition of TXNRD1 by Auranofin. AgomiR-NC, agomiR-21–3p and antagomiR-21–3p were all used at 100 nM in both cell lines. Erastin was used at 10 µM in both cell lines. RSL3 was used at 0.5 µM in A375 and 1 µM in A2058 cell line. Fer-1 was used at 2 µM in both cell lines. IFN-γ was used at 50 ng/mL in both cell lines. Auranofin was used at 2 µM in both cell lines. Data represent the mean±SD of triplicates. P value was calculated by two-tailed Student’s t-test. ^*^P<0.05, ^**^p<0.01, ^***^p<0.001. IFN, interferon; ns, non-significant; ROS, receiver operating characteristic; TXNRD1, thioredoxin reductase 1; 3’UTR, 3’ untranslated region.

We went on to identify the potential target of miR-21–3p that mediated its effect on lipid peroxidation in ferroptosis. We used publicly available bioinformatics tools TargetScan, miRWalk and miRanda to search for candidate genes containing potential miR-21–3p-binding sites in their 3’UTRs. TXNRD1 was of high predictive score and was selected out due to its pivotal role in the regulation of redox hemostasis through modulating a wide array of proteins with its selenocysteine-containing domain,^41^ whereas its effect on ferroptosis remains elusive. Besides, the 3’UTR region of TXNRD1 mRNA contained the putative miR-21–3p-binding site that is also highly conserved in many species (figure 3A). Subsequent firefly luciferase reporter assay showed that agomiR-21–3p significantly suppressed the luciferase activity in melanoma cells transfected with a plasmid harboring WT TXNRD1 3’UTR, but failed to influence the reporter activity in cells transfected with plasmids harboring mutant TXNRD1 3’UTR (figure 3B). To further confirm that whether TXNRD1 was truly a target of miR-21–3p, we examined the impact of miR-21–3p intervention on the mRNA and protein levels of TXNRD1. As a result, the overexpression of miR-21–3p via the treatment with agomiR-21-3p induced prominent downregulation of TXNRD1 in both mRNA and protein levels (figure 3C, D). In contrast, antagomiR-21-3p resulted in significant upregulation of TXNRD1 expression (figure 3C, D). These results demonstrated that TXNRD1 was a novel direct target of miR-21–3p in melanoma.

We then employed TXNRD1 inhibitor Auranofin to treat melanoma cells co-treated with antagomiR-21–3p and then underwent erastin or RSL3 stimulation. The inhibition of TXNRD1 activity by Auranofin abolished the protective effect of antagomiR-21–3p on erastin or RSL3-triggered ferroptosis in melanoma cells as revealed by CCK8 and colony formation assays (figures 2A and 3E, F). Congruently, intracellular MDA content and lipid ROS assayed by C11-BODIPY probe exhibited the same trend as that of cell ferroptosis (figure 3G, H, online supplemental figure S3A). Moreover, TXNRD1 inhibitor Auranofin could also restrain the suppressive function of antagomiR-21–3p in IFN-γ-potentiated ferroptosis as identified by CCK8 and colony formation assays (figures 2C and 3E,F), as well as the generation of intracellular MDA and lipid ROS level (figure 3G, H, online supplemental figure S3A). Taken together, miR-21–3p directly targeted TXNRD1 to promote lipid peroxidation generation and ferroptosis in melanoma.

### MiR-21-3p increases the efficacy of anti-PD-1 immunotherapy by promoting ferroptosis in melanoma

**Figure 4:**
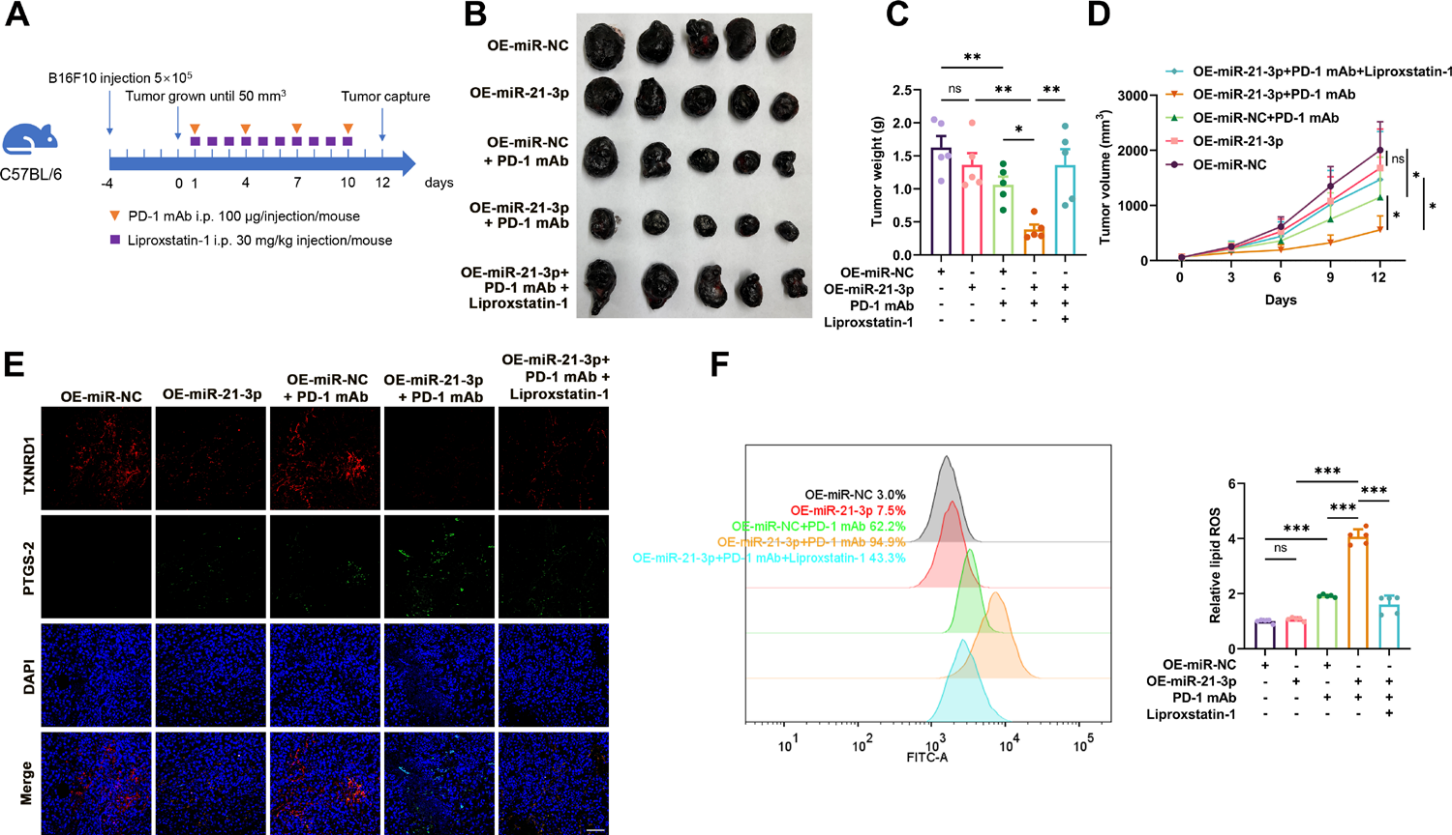
Tumorous miR-21–3p overexpression promotes the therapeutic efficacy of anti-PD-1 antibody by promoting ferroptosis. (A) A schematic view of the treatment plan that C57BL/6 mice burdened with B16F10 tumors with or without tumorous miR-21–3p overexpression received anti-PD-1 antibody and liprostatin-1 treatment as indicated. (B-D) Images of isolated tumors from mice that received indicated treatment. Tumor volumes and weights in each group were calculated and displayed in (C) and (D). (E) Immunofluorescence staining of PTGS2 and TXNRD1 in isolated transplanted tumors with indicated treatment. Scale bar=50 µm. (F) Relative lipid ROS in isolated transplanted tumors with indicated treatment. Data represent the mean±SD of triplicates. P value was calculated by two-tailed Student’s t-test. ^*^P<0.05, ^**^p<0.01, ^***^p<0.001. ns, non-significant; PD-1, programmed cell death protein 1; ROS, receiver operating characteristic; TXNRD1, thioredoxin reductase 1.

Given the prominent regulatory role of miR-21–3p in IFN-γ-potentiated ferroptosis, we supposed that increased miR-21–3p in tumor might contribute to the efficacy of anti-PD-1 immunotherapy via the regulation of tumor cell ferroptosis. To verify this, a preclinical transplanted tumor model was established via subcutaneous injection of B16F10 melanoma cells into the flank of C57BL/6 mice to evaluate the therapeutic effect of anti-PD-1 immunotherapy synergized with the overexpression of tumor miR-21–3p level obtained by the pretransfection with vector (figure 4A). The overexpression of miR-21–3p alone exerted no significant impact on tumor growth, whereas it could synergistically potentiate the tumor-suppressive effect of anti-PD-1 antibody on melanoma (figure 4B–D). Further immunofluorescence staining analysis of isolated transplanted tumors revealed that the staining intensity of PTGS2, an indicator of ferroptosis, was increased more prominently in the combined treatment group (figure 4E). In addition, flow cytometry using C11-BODIPY also revealed that lipid ROS generation was increased after anti-PD-1 antibody treatment and further potentiated after the overexpression of tumor miR-21–3p (figure 4F). Furthermore, the combined effect of anti-PD-1 antibody treatment and tumorous miR-21-3p overexpression on melanoma growth could be significantly blocked by the administration with liproxstatin-1 (figure 4B–F), indicating that the role of miR-21–3p overexpression in potentiating the efficacy of anti-PD-1 antibody depended on the induction of tumor cell ferroptosis. The immunofluorescence staining assay also showed that the expression of TXNRD1 was suppressed after miR-21–3p overexpression and after the combined treatment (figure 4E), supporting that TXNRD1 was the target of miR-21–3p in anti-PD-1 immunotherapy-associated ferroptosis *in vivo*.

### MiR-21-3p-loaded gold nanoparticle synergizes with anti-PD-1 immunotherapy in melanoma

**Figure 5:**
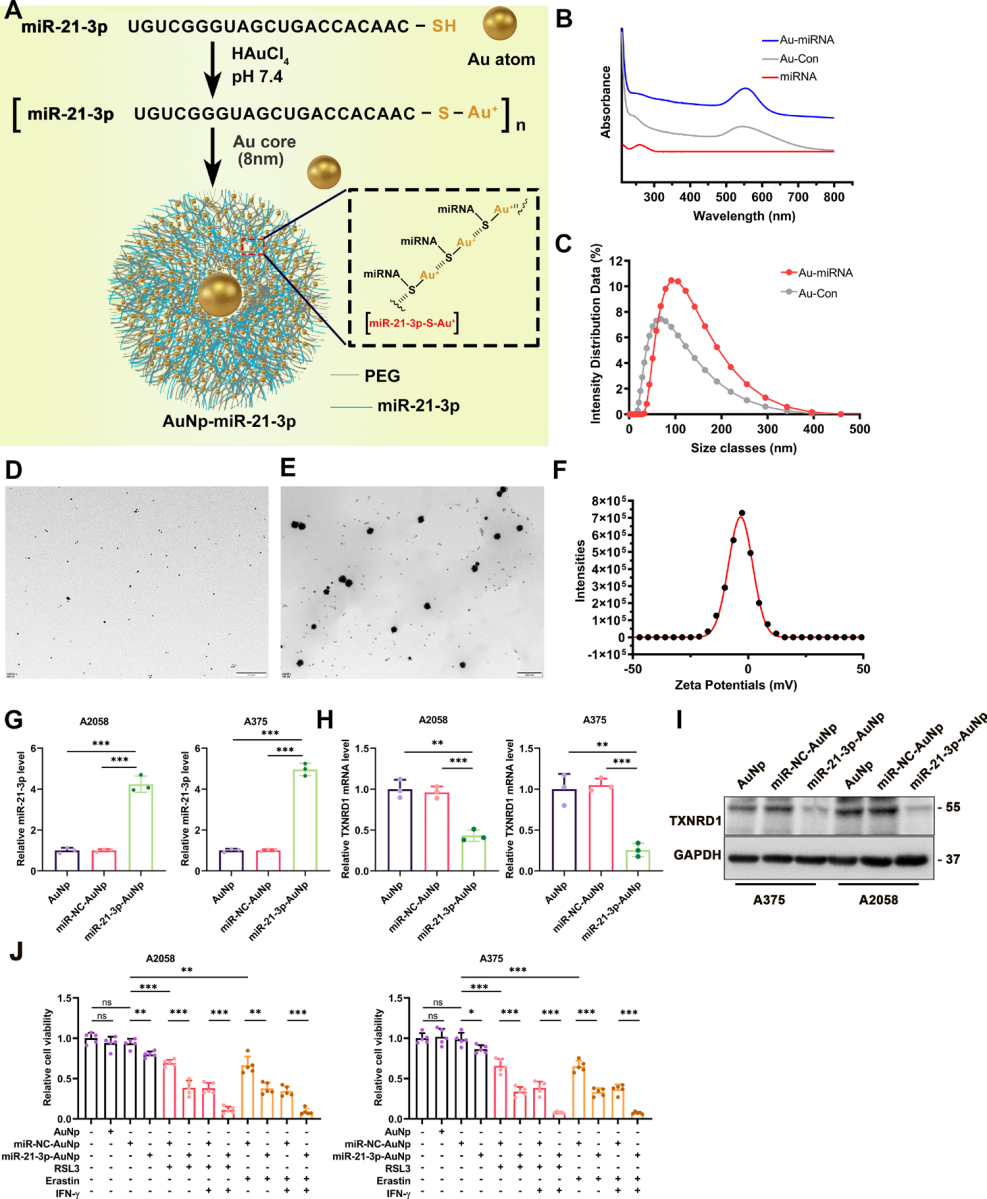
Synthesis and characterization of miR-21–3p-conjugated nanoparticles miR-21–3p-AuNp. (A) A schematic view of the design and construction process of miR-21–3p-AuNp. (B) UV-Vis absorption spectra of AuNp and miR-21–3p-AuNp. (C) Hydrodynamic diameter distributions of AuNp and miR-21–3p-AuNp, showing the successful self-assembly process of the nanoengineering miRNA into an auric sphere hybrid system. (D-E) Transmission electron micrograph images (TEM) of Au and miR-21–3p-AuNp. (F) The zeta potential of miR-21–3p-AuNp measured in PBS at pH 7.4. (G-H) Relative mRNA level of miR-21–3p and TXNRD1 after the treatment with miR-21–3p-AuNp or AuNp in melanoma cell. (I) Immunoblotting analysis of TXNRD1 after the treatment with miR-21–3p-AuNp or AuNp in melanoma cell. (J) Relative cell viability of melanoma cells treated with ferroptosis inducer, IFN-γ and miR-21–3p-AuNp or AuNp. Erastin was used at 10 µM in both cell lines. RSL3 was used at 0.5 µM in A375 and 1 µM in A2058 cell line. IFN-γ was used at 50 ng/mL in both cell lines. Data represent the mean±SD of triplicates. P value was calculated by two-tailed Student’s t-test. ^*^P<0.05, ^**^p<0.01, ^***^p<0.001. AuNp, gold nanoparticles; IFN, interferon; miRNA, microRNA; ns, non-significant; TXNRD1, thioredoxin reductase 1.

Previously, we reported the development of AuNp as a membrane-traversing delivery vehicle to carry peptide for anticancer therapy.^42^ This therapeutic approach could achieve prominent effects on tumor treatment with a favorable safety profile.^43^ Of note, some ongoing clinical trials employed nanoparticles to transfer drugs more precisely and efficiently to target tumor cells in cancer therapy (NCT03774680, NCT00629499). Regarding the overexpression of tumor miR-21–3p increased the efficacy of anti-PD-1 immunotherapy in the preclinical mouse model, we proposed that systemic delivery of miR-21–3p to tumor tissue by AuNp might be of high translational potential to synergize with anti-PD-1 immunotherapy. To synthesize miR-21–3p-loaded AuNp with high loading efficacy and stability, the antisense strand of miR-21–3p was modified with sulfhydryl (-SH), that is, miR-21–3p-SH, and reacted with HAuCl_4_ to form a polymeric miRNA-Au compound. With a reductive environment, Au^3+^ ions connected miR-21–3p-SH via the coordinate bond between Au and -SH. Furthermore, to potentiate miRNA loading, product stability and endosomal escape, thiol-PEG-amine (MW 2000 Da) was spiked at a molar ratio of 1:2 into miR-21–3p-SH for reaction with HAuCl_4_. Subsequent addition of 50 mM HEPES as a reductant and 1 mM seed AuNp (Au core) to the [miR-21–3p-S-Au1^+^]_n_ solution resulted in nanoparticles fabricated with miR-21–3p and PEG-amine, that is, miR-21–3p-AuNp (figure 5A). This high content of Au(I)/Au(0)-thiolate complexes was well proved by XPS as previously reported (figure 5A).^42^ The UV-Vis absorption spectra and hydrodynamic diameter distributions of AuNp and miR-21–3p-AuNp particles were also shown (figure 5B, C). In addition, miR-21–3p-AuNp was well-dispersed in solution as shown by TEM (figure 5D–E). Analysis of AuNp and miR-21–3p-AuNp by dynamic light scattering techniques revealed that nanohybrid growth of miRNA/PEGAu, under mildly reducing conditions, significantly increased the hydrodynamic diameter from ~70 to ~100 nm (figure 5C). The zeta potential of miR-21–3p-AuNp was nearly 0 mV (figure 5F). Treatment of melanoma cells with miR-21–3p-AuNp increased the intracellular level of miR-21–3p robustly compared with AuNp and miR-NC-AuNp treatment group (figure 5G). In parallel, the expression of TXNRD1 was significantly reduced in both mRNA and protein levels in A2058 and A375 cell lines (figure 5H, I). As a result, miR-21–3p-AuNp treatment could sensitize melanoma cells to ferroptosis induced by erastin/RSL3 monotreatment or with the combination of IFN-γ (figure 5J). These data indicated that nanoparticle-based transfection of melanoma cells could be used to increase miR-21–3p level to supraphysiological level and exert its regulatory role in ferroptosis.

To further determine whether systemic delivery of miR-21–3p by gold nanoparticle holds *in vivo* therapeutic potential to synergize with anti-PD-1 immunotherapy, the preclinical mice model established via the subcutaneous injection of B16F10 melanoma cells into the flank of C57BL/6 mice was employed, and anti-PD-1 antibody and miR-21–3p-loaded AuNp were both injected intraperitoneally as indicated (online supplemental figure S3B). First of all, we testified the cellular internalization status of miR-21–3p-AuNp *in vitro* and biodistribution *in vivo*. Flow cytometry analysis was employed to examine the cellular internalization status of sulfo-cyanine3 (Cy5)-conjugated miR-21–3p-AuNp by B16F10 melanoma cells and RAW264.7 macrophages, respectively. While Cy5-miR-21–3p-AuNp showed robust internalization into B16F10 melanoma cells (>80%), RAW264.7 macrophages took in much less Cy5-miR-21–3p-AuNp (around 35%) after the incubation for 6 hours, providing a highly favorable profile for circulation (online supplemental figure S3C). Furthermore, the biodistribution of miR-21–3p-AuNp in B16F10 transplanted melanoma mice model was examined by inductively coupled plasma mass spectrometry (ICP-MS), which was used to quantify ^197^Au concentrations in tissues and presented by the percentage of injected dose (ID) in per gram of organ or tumor (ID%/g) as previously described.^44 45^As was revealed, a time-dependent tendency for tumor accumulation can be found in the biodistribution results (online supplemental figure S3D). We also employed qRT-PCR analysis to examine the actual level of miR-21–3p in transplanted tumors and other normal organs *in vivo*. As a result, the level of miR-21–3p in tumor was robustly increased 4 hours after systemic delivery miR-21–3p-AuNp, and remained at a relatively high level until 72 hours postinjection (online supplemental figure S3E). Besides, the level of miR-21–3p in multiple normal organs including heart, liver, lung and kidney after systemic delivery miR-21–3p-AuNp displayed the alterations that were consistent with the results of ^197^Au concentrations detected by ICP-MS (online supplemental figure S3F). Of note, the mRNA level of TXNRD1 in tumor displayed prominent downregulation 24 hours after systemic delivery miR-21–3p-AuNp, and was at forwardly reduced 48 hours after treatment (online supplemental figure S3G). In addition, the protein level of TXNRD1 in tumor was also remarkably downregulated 48 hours after treatment (online supplemental figure S3G). These results suggested the very early miRNA delivery and efficient molecular knockdown effects already at days 1–2 after treatment.

**Figure 6:**
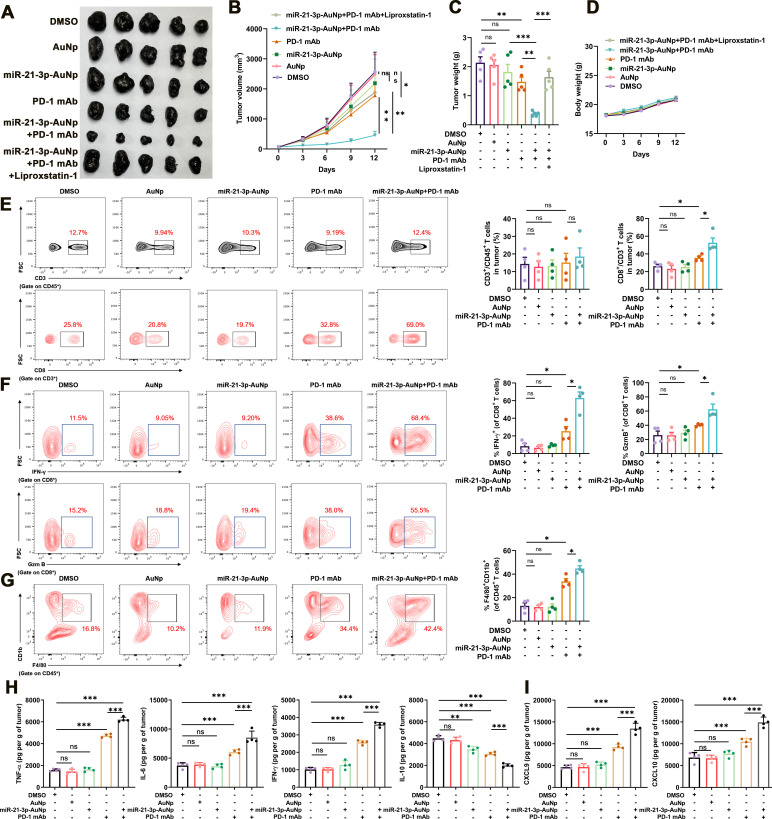
Nanoparticle delivery of miR-21–3p sensitizes melanoma to anti-PD-1 immunotherapy by promoting ferroptosis. (A-C) Images of isolated transplanted tumors from mice that received indicated treatment. Tumor volumes and weights in each group were calculated and displayed in (B) and (C). (D) Body weight of mice that received indicated treatment related to (A). (E) FACS of CD3^+^ in CD45^+^ cells and CD8^+^ in CD3^+^ TILs from B16F10 transplanted tumors with indicated treatment and the quantification. (F) FACS of IFN-γ^+^ and GzmB^+^ in CD8^+^ TILs from B16F10 transplanted tumors with indicated treatment and the quantification. (G) FACS of F4/80^+^CD11b^+^ in CD45^+^ TILs from B16F10 transplanted tumors with indicated treatment and the quantification. (H-I) Levels of cytokine and chemokines in tumors after indicated treatment. Data represent the mean±SD of triplicates. P value was calculated by two-tailed Student’s t-test. ^*^P<0.05, ^**^p<0.01, ^***^p<0.001. ns, non-significant; AuNp, gold nanoparticles; FACS, fluorescence-activated cell sorting; PD-1, programmed cell death protein 1; TIL, tumor infiltrating lymphocytes.

Subsequently, we testified the therapeutic effect of the combined treatment with anti-PD-1 antibody and miR-21–3p-AuNp. While the monotreatment with anti-PD-1 antibody resulted in a significant delay of tumor growth, the combination of miR-21–3p-AuNp and anti-PD-1 antibody could lead to more prominent delay of tumor progression as revealed by the alteration of tumor volume and tumor weight, which could be reversed by the administration with liproxstatin-1 (figure 6A–C). In addition, the body weights of mice after distinct indicated treatments were not significantly altered compared with the control group (figure 6D). Of note, monotreatment with AuNp exerted no therapeutic effect on tumor growth (figure 6A–C), supporting that the tumor suppressive role of miR-21–3p-AuNp was attributable solely to miR-21–3p delivery. Concurrent immunofluorescence staining in isolated transplanted tumors revealed that the intensity of PTGS2 was prominently increased in the combined treatment group, whereas the expression of TXNRD1 was attenuated more significantly in the combined treatment group compared with miR-21–3p-AuNp treatment group and anti-PD-1 antibody treatment group (online supplemental figure S4A). Furthermore, we went on to analyze the immune profile of transplanted B16F10 tumors after the combined treatment with miR-21–3p-AuNp and anti-PD-1 antibody, in particular the infiltration and cytotoxicity of CD8^+^ T cells, as well as the number of macrophages within TME, in another cohort of C57BL/6 mice. As a result, while the numbers of CD3^+^CD45^+^ lymphocytes within TME were comparable in different groups (figure 6E), the number of CD8^+^CD3^+^T cells was prominently increased after the treatment with anti-PD-1 antibody, and was forwardly increased after the combined treatment with miR-21–3p-AuNp (figure 6E). The antitumor capacity of tumor-infiltrating CD8^+^ T displayed similar trend as revealed by the positive percentage of granzyme B and IFN-γ in these cells in different groups (figure 6F). Furthermore, the combined treatment induced more infiltration of macrophages within TME, as revealed by the percentage of CD11b^+^F4/80^+^ in CD45^+^ lymphocytes (figure 6G). We also examined the content of some cytokines and chemokine in tumor that are crucial for antitumor immunity within TME via the employment of ELISA assay as previously described.^46^ It is revealed that the contents of TNF-α, IL-6 and IFN-γ were prominently increased after the treatment with anti-PD-1 antibody, and further potentiated after the combination with miR-21–3 p-AuNp. Conversely, the content of IL-10 that can suppress antitumor immune response was significantly decreased in combined treatment group (figure 6H). Furthermore, the chemokines CXCL9 and CXCL10 that are greatly implicated in the recruitment of CD8^+^ T cells to tumor were also upregulated after the treatment with anti-PD-1 antibody, and were further increased in response to the combined treatment (figure 6I). Therefore, miR-21–3p-AuNp co-treatment could facilitate the expression and secretion of pro-inflammatory cytokines and chemokines to enhance antitumor immunity. These findings all point out that the combination treatment strategy can amplify the antitumor immunity compared with anti-PD-1 antibody monotreatment.

To evaluate the role of miR-21–3p-AuNp treatment in systemic immunity and the toxicity *in vivo*, we measured the level of serum IL-2, IFN-γ, TNF-α and IL-6, as well as eosinophil and erythropoietin in the blood in response to miR-21–3 p-AuNp administration as previously demonstrated,^43^ all of which showed no obvious changes compared with the control (online supplemental figure S4B), indicating the hypo-immunogenicity of miR-21–3p-AuNp when systemically delivered. The safety of miR-21–3p-AuNp was further confirmed by the steady-state in the number of white blood cells, thrombocytes, red blood cells and hemoglobin after 12 days’ treatment (online supplemental figure S4C). H&E staining of the liver, spleen, kidney, heart and lung also revealed no prominent pathological dysregulation and supported the conclusion that miR-21–3p-AuNp was sufficiently safe when systemically administrated (online supplemental figure S4D).

In order to rule out the possibility that the therapeutic effect of combined anti-PD-1 antibody and miR-21–3p-AuNp is related to a direct effect on melanoma cells whereas not mediated by the immune system, we have performed animal experiments in C57BL/6 mice in which CD8^+^ T cells are systemically eliminated by intraperitoneal CD8α antibody injection (online supplemental figure S4E). As a result, systemic CD8α antibody treatment could significantly reverse the effect of the combination of anti-PD-1 antibody and miR-21–3p-AuNp (online supplemental figure S4F-H), indicating that synergized effect was highly dependent on immune system and the actual involvement of T cells in antitumor synergy. This result is consistent with the previous report that the induction of tumor cell ferroptosis is mediated by IFN-γ derived from tumor-infiltrating CD8^+^ T cells within TME. Therefore, the therapeutic effect of the combination of miR-21–3p-AuNp and anti-PD-1 antibody was attributed to the CD8^+^ T cells-dependent antitumor immunity and IFN-γ-driven tumor cell ferroptosis.

### ATF3 transcriptionally upregulates miR-21-3p in IFN-γ-stimulated ferroptosis

**Figure 7:**
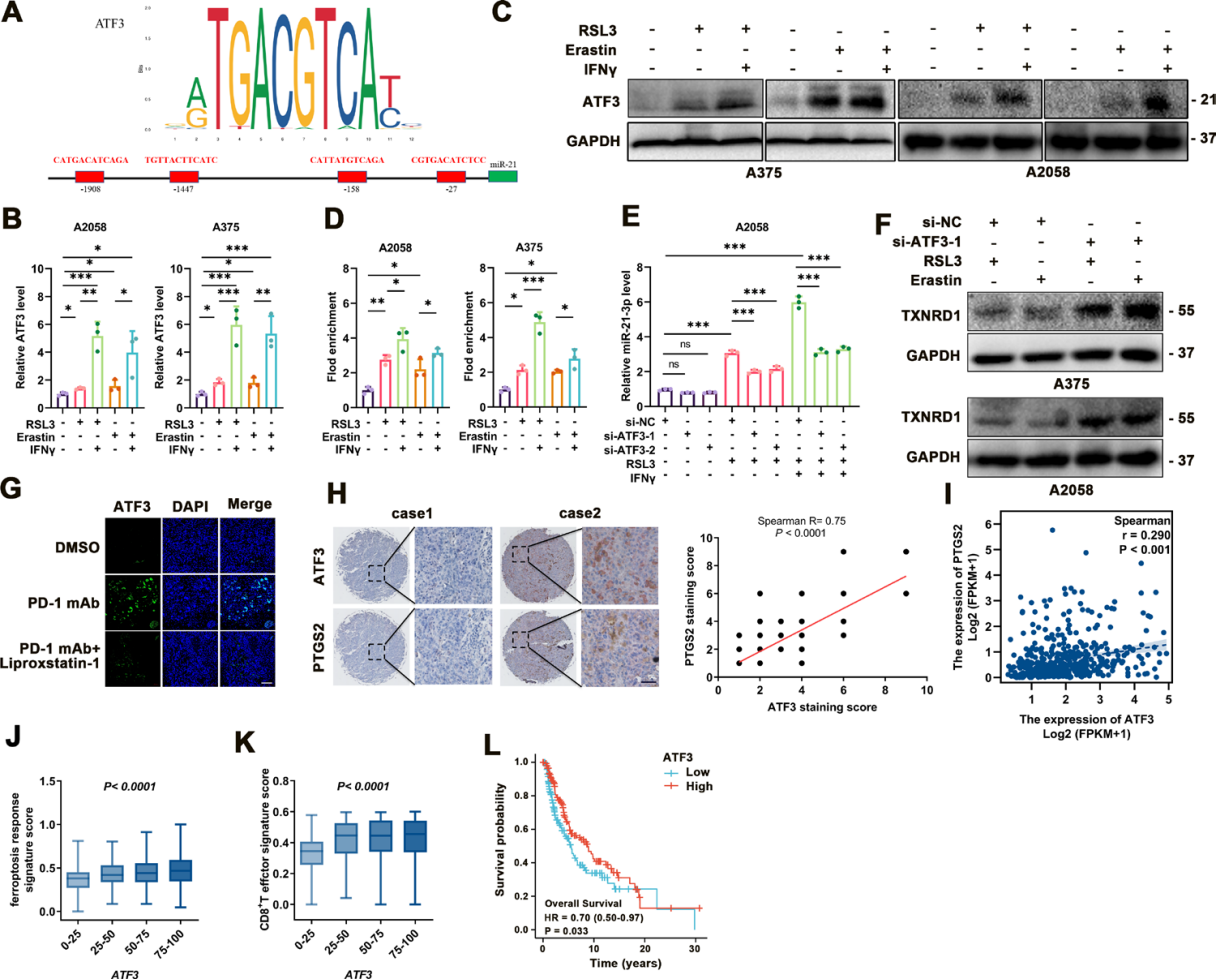
ATF3 upregulation contributes to the increase of miR-21–3p in ferroptosis. (A) Transcription factor analysis of miR-21–3p promoter identified four ATF3 binding sites. (B-C) mRNA and protein levels of ATF3 in response to IFN-γ-potentiated ferroptosis. (D) Chromatin immunoprecipitation analysis of the enrichment of ATF3 to the promoter of miR-21–3p in IFN-γ-potentiated ferroptosis. (E) Relative level of miR-21–3p in IFN-γ-potentiated ferroptosis with or without the knockdown of ATF3. (F) Protein levels of TXNRD1 in response to ferroptosis inducers with or without the knockdown of ATF3. (G) Immunofluorescence staining of ATF3 in isolated transplanted tumors with indicated treatment. Scale bar=50 µm. (H) Immunohistochemical staining analysis of ATF3 and PTGS2 in TMA, with the correlation of staining scores displaying on the right. (I) The correlation between ATF3 and PTGS2 mRNA level in TCGA SKCM database. (J-K) The correlation between ATF3 mRNA level and ferroptosis response score or CD8^+^ T effector signature score in TCGA SKCM database. (L) Kaplan-Meier survival analysis of patients with melanoma between high tumorous ATF3 and low tumorous ATF3 level defined by the median level. Erastin was used at 10 µM in both cell lines. RSL3 was used at 0.5 µM in A375 and 1 µM in A2058 cell line. IFN-γ was used at 50 ng/mL in both cell lines. Data represent the mean±SD of triplicates. P value was calculated by two-tailed Student’s t-test. ^**^P<0.01, ^***^p<0.001. DMSO, dimethyl sulfoxide; IFN, interferon; ns, non-significant; TMA, tumor tissue microarray; TXNRD1, thioredoxin reductase 1.

Ultimately, we wondered the regulatory mechanism responsible for the upregulation of miR-21–3p in melanoma ferroptosis. To this end, hTFtarget analysis illustrated the potential binding sites of several transcriptional factors to the promoter of miR-21–3p, and we noticed that ATF3 was the candidate upstream regulator (figure 7A). ATF3 was reportedly a versatile transcriptional factor involved in multiple biological processes like cell death.^47–50^ More importantly, as a common stress sensor, ATF3 could directly bind to the promoter of SLC7A11 to suppress its transcription and thereby result in lipid peroxidation and ferroptosis after the stimulation with erastin.^50^ Consistent with previous reports, our data showed that both the mRNA and protein levels of ATF3 were significantly increased in response to either erastin/RSL3 monotreatment, and were further upregulated after combined treatment with IFN-γ in melanoma cells (figure 7B, C).

Chromatin immunoprecipitation assay showed more enrichment of ATF3 to the promoter of miR-21–3p on the treatment with erastin/RSL3, which was forwardly potentiated after combined treatment with IFN-γ (figure 7D). In addition, ATF3 knockdown was found to prominently abolish the upregulation of miR-21–3p in melanoma cells after either monotreatment with RSL3 or combined with IFN-γ (figure 7E). In accordance, the knockdown of ATF3 could increase the expression of TXNRD1 under the treatment with erastin or RSL3 (figure 7F). Further immunofluorescence staining analysis suggested that on anti-PD-1 antibody treatment, the staining intensity of ATF3 was prominently increased, and co-administration of liproxstatin-1 in vivo reduced the staining intensity of ATF3 (figure 7G).

We then employed immunohistochemical staining of TMA consisting of 82 melanoma tissues to analyze the relationship between ATF3 and ferroptosis indicator PTGS2, which revealed a significant positive correlation between the staining scores of them (figure 7H). We also turned to TCGA SKCM database to investigate the clinical implications of ATF3 in melanoma and the relationship between ATF3 expression and ferroptosis and antitumor immunity. As was shown, the mRNA level of ATF3 was in positive correlation with ferroptosis indicator PTGS2 (figure 7I). Moreover, ATF3 mRNA level was also positively correlated with ferroptosis response signature score and CD8^+^ T effector signature score, respectively (figure 7J–K). More importantly, high expression of ATF3 significantly correlated with better survival of patients with melanoma (figure 7L). Taken together, these results demonstrated the crucial role of ATF3 in promoting miR-21–3p upregulation in IFN-γ-potentiated ferroptosis and its implication in melanoma pathogenesis and anti-PD-1 immunotherapy.

## Discussion

Ferroptosis is a unique cell death modality that differs from apoptosis or necroptosis, and is characterized by iron-dependent excessive generation of lipid peroxidation.^51^ Previous studies in terms of cancer treatment mainly emphasized the role of cell apoptosis.^6 7^ However, anti-PD-1 antibody immunotherapy could activate tumor-infiltrating CD8^+^ T cells to secret IFN-γ, which suppressed the expression of glutamate-cystine antiporter system Xc^−^ and triggered ferroptosis of melanoma cells.^8–10^ Herein, we proved that the inhibition of ferroptosis prominently dampened the efficacy of anti-PD-1 antibody. Therefore, to find novel regulatory molecules of ferroptosis-promotive function can provide novel synergistic targets to sensitize melanoma cells to immunotherapy. Jiang *et al* recently reported that tumors with high TYRO3 expression exhibited anti-PD-1/PD-L1 resistance in a syngeneic mouse model and in patients who received anti-PD-1/PD-L1 therapy partially due to its suppressive effect on tumor cell ferroptosis. Arguably, TYRO3 could serve as a predictive biomarker for patient selection and a promising therapeutic target to overcome anti-PD-1/PD-L1 resistance.^52^ Similarly, our data illustrated the regulatory mechanism of melanoma cell ferroptosis from the perspective of miRNAs modulation by obtaining the expression profile of miRNAs. Some previous studies have yielded therapeutic insights into the regulation of ferroptosis by miRNA in melanoma. In particular, miR-9 reportedly regulated ferroptosis by targeting glutamic-oxaloacetic transaminase GOT1 in melanoma.^53^ Besides, miR-137 could negatively regulate ferroptosis by directly targeting glutamine transporter SLC1A5 in melanoma cells.^17^ Supplementary to these, our results illustrated that miR-21–3p upregulation contributed to IFN-γ-potentiated ferroptosis by directly targeting TXNRD1 and promoting lipid peroxidation, thus extending the regulatory network of ferroptosis-associated miRNAs and providing novel targets to increase the efficacy of immunotherapy. Further investigations could be employed to find more candidate miRNAs with competent translational potential in melanoma therapy.

MiRNAs are an abundant class of small non-coding RNAs that negatively modulate gene expression by targeting 3’UTR of mRNAs.^54^ We for the first time found that TXNRD1 was the novel target of miR-21–3p and mediated its role in ferroptosis with a specific impact on lipid peroxidation. TXNRD1 is the rate-limiting enzyme in the thioredoxin anti-oxidant pathway.^55^ A recent study has reported that TXNRD1 activation played a protective role in lysine oxidase-triggered ferroptosis, and the inhibition of TXNRD1 could enhance the cytotoxic effect of lysine oxidase prominently.^56^ In line with this, our data also supported that TXNRD1 could guard against ferroptosis via ameliorating lipid peroxidation. Therefore, to intervene the upstream regulators of TXNRD1 like miR-21–3p could be promising in triggering tumor cell ferroptosis and increasing the efficacy of immunotherapy.

There are two mature forms of miR-21, namely, miR-21–5p and miR-21–3p, and miR-21 commonly refers to miR-21–5p according to the information of sequence and IDs in MiRBase website (https://www.mirbase.org/). Previous studies have demonstrated that miR-21 (namely miR-21–5p) is an oncogenic factor that can promote cell proliferation and metastasis and suppress cell apoptosis in melanoma.^57–60^ In addition, miR-21–5p has been proposed as a plausible diagnostic and prognostic biomarker, as well as a therapeutic target for several types of cancers.^61^ Nevertheless, the actual role of miR-21–3p in melanoma pathogenesis remains elusive. According to our results, ectopic expression of miR-21–3p induced a slight but significant reduction of both cell viability and colony formation *in vitro*. However, there was no significant alteration of tumor growth in miR-21–3p-AuNp treatment group compared with AuNp treatment group. Therefore, the antitumor effect of miR-21–3p *in vivo* was merely attributed to its role in melanoma cell proliferation. Instead, the synergized therapeutic effect of combining miR-21–3p-AuNp and anti-PD-1 antibody was dependent on the induction of tumor cell ferroptosis triggered by tumor-infiltrating T cells. Besides, since that miR-21–3 p alone exerted suppressive role in melanoma cell proliferation in vitro whereas insignificant effect on tumor growth in vivo, it is of low possibility that nanoparticle delivery of miR-21–3 p might paradoxically lead to tumor expansion rather than control. Based on our findings, the combinatorial treatment strategy could be applied to increase the sensitivity to immune checkpoint inhibitors in immunogenic melanomas that already harbor abundant infiltrating CD8+ T cells, especially for those that might be adaptively resistant to anti-PD-1 immunotherapy. The lack of T cell response within TME could probably limit the efficacy of this strategy. Therefore, the status of infiltrating CD8+ T cells within TME should be taken into account when determining the clinical implication of this combinatorial treatment strategy.

ATF3 is a versatile transcriptional factor implicated in various biological activities including cell proliferation, stress response, metabolism and antitumor immunity.^47 62–64^ As a common stress sensor, ATF3 facilitates ferroptosis by potentiating lipid peroxidation via transcriptionally activating miR-21–3 p in addition to the regulation of cystine uptake.^13^ In line with this report, our results also demonstrated that ATF3 expression was increased under RSL3/erastin treatment or the combined stimulation with IFN-γ. Moreover, ATF3 could favor ferroptosis by potentiating lipid peroxidation via transcriptionally activating miR-21–3 p in addition to the regulation of cystine uptake,^13^ indicating the co-existence of multiple regulatory mechanisms responsible for the crucial effect of ATF3. More importantly, Liu *et al* reported that the induction of ATF3 after ADORA1 inhibition promoted the expression of PD-L1 to suppress the activity of tumor-infiltrating lymphocytes and thus increased the efficacy of anti-PD-1 immunotherapy.^65^ Our present results indicated that ATF3 might contribute to the enhanced effect of anti-PD-1 antibody by facilitating tumor cell ferroptosis, in addition to the effect on immune checkpoint molecules and antitumor immunity. Therefore, ATF3 could act as a potent modulator of immunotherapy by simultaneously affecting the tumor cell itself and the tumor microenvironment.

Various types of nanoparticles like micelles, liposomes, polymers and AuNp have been widely used as delivery vehicles for miRNAs and peptides to target tumor mRNAs and protein-protein interaction for cancer therapy.^66–71^ Of note, AuNp is characterized by chemical inertness, biocompatibility, convenience in preparation and relative high cellular uptake efficiency,^72 73^ and accumulative clinical trials revealed that miRNAs-based mimic drugs carried by multiple carriers had potent antitumor capacity with high specificity targeting tumor cells and some patients receiving these treatments gained encouraging response (NCT04675996).^23–25^ Our data proved that systemic delivery of miR-21–3 p by AuNp could robustly increase the level of miR-21–3 p in transplanted tumors. More importantly, we also confirmed the low toxicity of miR-21–3 p-AuNp with the steady state in the number of blood cells and the maintenance of the physiological structure of multiple crucial organs after systemic administration. The low toxicity results from the rapid clearance of nanoparticles from normal organs and reduced propensity to aggregate that was highly associated with its toxicity and ameliorated therapeutic efficacy.^42^ Additionally, our results proved that nanoparticle delivery of miR-21–3 p could sensitize melanoma cells to anti-PD-1 immunotherapy by facilitating ferroptosis, highlighting it as a novel therapeutic approach to synergize with immunotherapy. Further investigations including preclinical experiments and clinical trials are needed to evaluate its translational potential in the future.

## Conclusion

In the present study, our data demonstrate that ATF3-induced miR-21–3 p upregulation contributed to the efficacy of anti-PD-1 immunotherapy by facilitating tumor cell ferroptosis via the suppression of the novel target TXNRD1 and lipid peroxidation. Nanoparticle delivery of miR-21–3 p could be exploited as a promising therapeutic approach to increase the efficacy of immunotherapy without prominent systemic side effects, which warrants further investigations in future clinical trials.

## Data Availability

Sequencing data is accessible through GEO series accession numbers GSE186497. Source data for Figures are available from the corresponding authors upon request.

## References

[1] Lo JA, Fisher DE. The melanoma revolution: from UV carcinogenesis to a new era in therapeutics. Science 2014;346:945–9. 10.1126/science.1253735 25414302 PMC4701046

[2] Curti BD, Faries MB. Recent advances in the treatment of melanoma. N Engl J Med 2021;384:2229–40. 10.1056/NEJMra2034861 34107182

[3] Weiss SA, Wolchok JD, Sznol M. Immunotherapy of melanoma: facts and hopes. Clin Cancer Res 2019;25:5191–201. 10.1158/1078-0432.CCR-18-1550 30923036 PMC6726509

[4] Yarchoan M, Hopkins A, Jaffee EM. Tumor mutational burden and response rate to PD-1 inhibition. N Engl J Med 2017;377:2500–1. 10.1056/NEJMc1713444 29262275 PMC6549688

[5] Pitt JM, Vétizou M, Daillère R, et al. Resistance mechanisms to immune-checkpoint blockade in cancer: tumor-intrinsic and -extrinsic factors. Immunity 2016;44:1255–69. 10.1016/j.immuni.2016.06.001 27332730

[6] Wang Y, Li J-J, Ba H-J, et al. Down Regulation of c-FLIP_L_ Enhance PD-1 Blockade Efficacy in B16 Melanoma. Front Oncol 2019;9:857. 10.3389/fonc.2019.00857 31552181 PMC6738195

[7] Hendriks D, He Y, Koopmans I, et al. Programmed Death Ligand 1 (PD-L1)-targeted TRAIL combines PD-L1-mediated checkpoint inhibition with TRAIL-mediated apoptosis induction. Oncoimmunology 2016;5:e1202390. 10.1080/2162402X.2016.1202390 27622071 PMC5007955

[8] Zitvogel L, Kroemer G. Interferon-γ induces cancer cell ferroptosis. Cell Res 2019;29:692–3. 10.1038/s41422-019-0186-z 31160718 PMC6796847

[9] Wang W, Green M, Choi JE, et al. CD8^+^ T cells regulate tumour ferroptosis during cancer immunotherapy. Nature 2019;569:270–4. 10.1038/s41586-019-1170-y 31043744 PMC6533917

[10] Lang X, Green MD, Wang W, et al. Radiotherapy and immunotherapy promote tumoral lipid oxidation and ferroptosis via synergistic repression of Slc7a11. Cancer Discov 2019;9:1673–85. 10.1158/2159-8290.CD-19-0338 31554642 PMC6891128

[11] Cramer SL, Saha A, Liu J, et al. Systemic depletion of L-cyst(e)ine with cyst(e)inase increases reactive oxygen species and suppresses tumor growth. Nat Med 2017;23:120–7. 10.1038/nm.4232 27869804 PMC5218918

[12] Zhu S, Zhang Q, Sun X, et al. HSPA5 regulates Ferroptotic cell death in cancer cells. Cancer Res 2017;77:2064–77. 10.1158/0008-5472.CAN-16-1979 28130223 PMC5392369

[13] Wang L, Liu Y, Du T, et al. ATF3 promotes erastin-induced ferroptosis by suppressing system Xc.. Cell Death Differ 2020;27:662–75. 10.1038/s41418-019-0380-z 31273299 PMC7206049

[14] Gebert LFR, MacRae IJ. Regulation of microRNA function in animals. Nat Rev Mol Cell Biol 2019;20:21–37. 10.1038/s41580-018-0045-7 30108335 PMC6546304

[15] Zhang X, Wang L, Li H, et al. Crosstalk between noncoding RNAs and ferroptosis: new dawn for overcoming cancer progression. Cell Death Dis 2020;11:580. 10.1038/s41419-020-02772-8 32709863 PMC7381619

[16] Xie B, Guo Y. Molecular mechanism of cell ferroptosis and research progress in regulation of ferroptosis by noncoding RNAs in tumor cells. Cell Death Discov 2021;7:101. 10.1038/s41420-021-00483-3 33980834 PMC8115351

[17] Luo M, Wu L, Zhang K, et al. miR-137 regulates ferroptosis by targeting glutamine transporter SLC1A5 in melanoma. Cell Death Differ 2018;25:1457–72. 10.1038/s41418-017-0053-8 29348676 PMC6113319

[18] Zhang H, Deng T, Liu R, et al. CAF secreted miR-522 suppresses ferroptosis and promotes acquired chemo-resistance in gastric cancer. Mol Cancer 2020;19:43. 10.1186/s12943-020-01168-8 32106859 PMC7045485

[19] Xue C, Hu S, Gao Z-H, et al. Programmably tiling rigidified DNA brick on gold nanoparticle as multi-functional shell for cancer-targeted delivery of siRNAs. Nat Commun 2021;12:2928. 10.1038/s41467-021-23250-5 34006888 PMC8131747

[20] Revia RA, Stephen ZR, Zhang M. Theranostic nanoparticles for RNA-based cancer treatment. Acc Chem Res 2019;52:1496–506. 10.1021/acs.accounts.9b00101 31135134 PMC6701180

[21] Xin Y, Huang M, Guo WW, et al. Nano-based delivery of RNAi in cancer therapy. Mol Cancer 2017;16:134. 10.1186/s12943-017-0683-y 28754120 PMC5534073

[22] Fernandez-Piñeiro I, Badiola I, Sanchez A. Nanocarriers for microRNA delivery in cancer medicine. Biotechnol Adv 2017;35:350–60. 10.1016/j.biotechadv.2017.03.002 28286148

[23] Hong DS, Kang Y-K, Borad M, et al. Phase 1 study of MRX34, a liposomal miR-34a mimic, in patients with advanced solid tumours. Br J Cancer 2020;122:1630–7. 10.1038/s41416-020-0802-1 32238921 PMC7251107

[24] Telford BJ, Yahyanejad S, de Gunst T, et al. Multi-modal effects of 1B3, a novel synthetic miR-193a-3p mimic, support strong potential for therapeutic intervention in oncology. Oncotarget 2021;12:422–39. 10.18632/oncotarget.27894 33747358 PMC7939530

[25] Beg MS, Brenner AJ, Sachdev J, et al. Phase I study of MRX34, a liposomal miR-34a mimic, administered twice weekly in patients with advanced solid tumors. Invest New Drugs 2017;35:180–8. 10.1007/s10637-016-0407-y 27917453 PMC5893501

[26] van Zandwijk N, Pavlakis N, Kao SC, et al. Safety and activity of microRNA-loaded minicells in patients with recurrent malignant pleural mesothelioma: a first-in-man, phase 1, open-label, dose-escalation study. Lancet Oncol 2017;18:1386–96. 10.1016/S1470-2045(17)30621-6 28870611

[27] Shi Q, Zhang W, Guo S, et al. Oxidative stress-induced overexpression of miR-25: the mechanism underlying the degeneration of melanocytes in vitiligo. Cell Death Differ 2016;23:496–508. 10.1038/cdd.2015.117 26315342 PMC5072443

[28] Wu Z, Jia J, Xu X, et al. Human herpesvirus 6A promotes glycolysis in infected T cells by activation of mTOR signaling. PLoS Pathog 2020;16:e1008568. 10.1371/journal.ppat.1008568 32516328 PMC7282626

[29] Guo W, Ma J, Yang Y, et al. ATP-Citrate lyase epigenetically potentiates oxidative phosphorylation to promote melanoma growth and adaptive resistance to MAPK inhibition. Clin Cancer Res 2020;26:2725–39. 10.1158/1078-0432.CCR-19-1359 32034077

[30] Rosenberg JE, Hoffman-Censits J, Powles T, et al. Atezolizumab in patients with locally advanced and metastatic urothelial carcinoma who have progressed following treatment with platinum-based chemotherapy: a single-arm, multicentre, phase 2 trial. Lancet 2016;387:1909–20. 10.1016/S0140-6736(16)00561-4 26952546 PMC5480242

[31] Dixon SJ, Patel DN, Welsch M, et al. Pharmacological inhibition of cystine-glutamate exchange induces endoplasmic reticulum stress and ferroptosis. Elife 2014;3:e02523. 10.7554/eLife.02523 24844246 PMC4054777

[32] Kim Y, Bismeijer T, Zwart W, et al. Genomic data integration by WON-PARAFAC identifies interpretable factors for predicting drug-sensitivity in vivo. Nat Commun 2019;10:5034. 10.1038/s41467-019-13027-2 31695042 PMC6834616

[33] Tao N, Li K, Liu J, et al. Liproxstatin-1 alleviates bleomycin-induced alveolar epithelial cells injury and mice pulmonary fibrosis via attenuating inflammation, reshaping redox equilibrium, and suppressing ROS/p53/α-SMA pathway. Biochem Biophys Res Commun 2021;551:133–9. 10.1016/j.bbrc.2021.02.127 33735625

[34] Liu J, Kang R, Tang D. Signaling pathways and defense mechanisms of ferroptosis. Febs J 2021;41. doi:10.1111/febs.16059. [Epub ahead of print: 06 Jun 2021]. 34092035

[35] Kowsari K, Lee W, Yoo S-S, et al. Scalable visible light 3D printing and bioprinting using an organic light-emitting diode microdisplay. iScience 2021;24:103372. 10.1016/j.isci.2021.103372 34825139 PMC8605192

[36] Lei G, Mao C, Yan Y, et al. Ferroptosis, radiotherapy, and combination therapeutic strategies. Protein Cell 2021;12:836–57. 10.1007/s13238-021-00841-y 33891303 PMC8563889

[37] Zhao T, Guo X, Sun Y. Iron accumulation and lipid peroxidation in the aging retina: implication of ferroptosis in age-related macular degeneration. Aging Dis 2021;12:529–51. 10.14336/AD.2020.0912 33815881 PMC7990372

[38] Homma T, Fujii J. Application of glutathione as anti-oxidative and anti-aging drugs. Curr Drug Metab 2015;16:560–71. 10.2174/1389200216666151015114515 26467067

[39] Sucker A, Zhao F, Pieper N, et al. Acquired IFNγ resistance impairs anti-tumor immunity and gives rise to T-cell-resistant melanoma lesions. Nat Commun 2017;8:15440. 10.1038/ncomms15440 28561041 PMC5460020

[40] Ghandi M, Huang FW, Jané-Valbuena J, et al. Next-generation characterization of the cancer cell line encyclopedia. Nature 2019;569:503–8. 10.1038/s41586-019-1186-3 31068700 PMC6697103

[41] Wright DE, Altaany Z, Bi Y, et al. Acetylation regulates thioredoxin reductase oligomerization and activity. Antioxid Redox Signal 2018;29:377–88. 10.1089/ars.2017.7082 29117711 PMC6025699

[42] He W, Yan J, Li Y, et al. Resurrecting a p53 peptide activator - An enabling nanoengineering strategy for peptide therapeutics. J Control Release 2020;325:293–303. 10.1016/j.jconrel.2020.06.041 32653500

[43] She J, Li Y, Yan S, et al. De novo supraparticle construction by a self-assembled Janus cyclopeptide to tame hydrophilic microRNA and hydrophobic molecule for anti-tumor cocktail therapy and augmented immunity. Chem Eng J 2020;401:126080. 10.1016/j.cej.2020.126080

[44] Li L, He W, You W, et al. Turing miRNA into infinite coordination supermolecule: a general and enabling nanoengineering strategy for resurrecting nuclear acid therapeutics. J Nanobiotechnology 2022;20:10. 10.1186/s12951-021-01212-9 34983557 PMC8725389

[45] Yan S, Yan J, Liu D, et al. A nano-predator of pathological MDMX construct by clearable supramolecular gold(I)-thiol-peptide complexes achieves safe and potent anti-tumor activity. Theranostics 2021;11:6833–46. 10.7150/thno.59020 34093856 PMC8171083

[46] Rao L, Wu L, Liu Z, et al. Hybrid cellular membrane nanovesicles amplify macrophage immune responses against cancer recurrence and metastasis. Nat Commun 2020;11:4909. 10.1038/s41467-020-18626-y 32999291 PMC7527506

[47] Di Marcantonio D, Martinez E, Kanefsky JS, et al. ATF3 coordinates serine and nucleotide metabolism to drive cell cycle progression in acute myeloid leukemia. Mol Cell 2021;81:2752–64. 10.1016/j.molcel.2021.05.008 34081901 PMC8452149

[48] Duncan RM, Reyes L, Moats K, et al. ATF3 coordinates antitumor synergy between epigenetic drugs and protein disulfide isomerase inhibitors. Cancer Res 2020;80:3279–91. 10.1158/0008-5472.CAN-19-4046 32561529 PMC7442646

[49] Shi Z, Diao D, Zhao Y, et al. C/EBP homologous protein deficiency enhances hematopoietic stem cell function via reducing ATF3/ROS-induced cell apoptosis. Aging Cell 2021;20:e13382. 10.1111/acel.13382 34128315 PMC8282275

[50] Lu S, Wang X-Z, He C, et al. Atf3 contributes to brucine-triggered glioma cell ferroptosis via promotion of hydrogen peroxide and iron. Acta Pharmacol Sin 2021;42:1690–702. 10.1038/s41401-021-00700-w 34112960 PMC8463534

[51] Stockwell BR, Friedmann Angeli JP, Bayir H, et al. Ferroptosis: a regulated cell death nexus linking metabolism, redox biology, and disease. Cell 2017;171:273–85. 10.1016/j.cell.2017.09.021 28985560 PMC5685180

[52] Jiang Z, Lim S-O, Yan M, et al. Tyro3 induces anti-PD-1/PD-L1 therapy resistance by limiting innate immunity and tumoral ferroptosis. J Clin Invest 2021;131:e139434. 10.1172/JCI139434 PMC8262501 33855973

[53] Zhang K, Wu L, Zhang P, et al. miR-9 regulates ferroptosis by targeting glutamic-oxaloacetic transaminase GOT1 in melanoma. Mol Carcinog 2018;57:1566–76. 10.1002/mc.22878 30035324

[54] Bracken CP, Scott HS, Goodall GJ. A network-biology perspective of microRNA function and dysfunction in cancer. Nat Rev Genet 2016;17:719–32. 10.1038/nrg.2016.134 27795564

[55] Busker S, Page B, Arnér ESJ. To inhibit TrxR1 is to inactivate STAT3-Inhibition of TrxR1 enzymatic function by STAT3 small molecule inhibitors. Redox Biol 2020;36:101646. 10.1016/j.redox.2020.101646 32863208 PMC7378686

[56] Chepikova OE, Malin D, Strekalova E, et al. Lysine oxidase exposes a dependency on the thioredoxin antioxidant pathway in triple-negative breast cancer cells. Breast Cancer Res Treat 2020;183:549–64. 10.1007/s10549-020-05801-4 32696316 PMC7501192

[57] Yang CH, Yue J, Pfeffer SR, et al. Microrna miR-21 regulates the metastatic behavior of B16 melanoma cells. J Biol Chem 2011;286:39172–8. 10.1074/jbc.M111.285098 21940630 PMC3234742

[58] Satzger I, Mattern A, Kuettler U, et al. microRNA-21 is upregulated in malignant melanoma and influences apoptosis of melanocytic cells. Exp Dermatol 2012;21:509–14. 10.1111/j.1600-0625.2012.01510.x 22716245

[59] Martin del Campo SE, Latchana N, Levine KM, et al. MiR-21 enhances melanoma invasiveness via inhibition of tissue inhibitor of metalloproteinases 3 expression: in vivo effects of miR-21 inhibitor. PLoS One 2015;10:e0115919. 10.1371/journal.pone.0115919 25587717 PMC4294659

[60] Mao X-H, Chen M, Wang Y, et al. MicroRNA-21 regulates the ERK/NF-κB signaling pathway to affect the proliferation, migration, and apoptosis of human melanoma A375 cells by targeting SPRY1, PDCD4, and PTEN. Mol Carcinog 2017;56:886–94. 10.1002/mc.22542 27533779

[61] Bautista-Sánchez D, Arriaga-Canon C, Pedroza-Torres A, et al. The promising role of miR-21 as a cancer biomarker and its importance in RNA-based therapeutics. Mol Ther Nucleic Acids 2020;20:409–20. 10.1016/j.omtn.2020.03.003 32244168 PMC7118281

[62] Chen W-J, Lai Y-J, Lee J-L, et al. CREB/ATF3 signaling mediates indoxyl sulfate-induced vascular smooth muscle cell proliferation and neointimal formation in uremia. Atherosclerosis 2020;315:43–54. 10.1016/j.atherosclerosis.2020.11.009 33227547

[63] Liu Y, Hu Y, Xiong J, et al. Overexpression of activating transcription factor 3 alleviates cardiac microvascular ischemia/reperfusion injury in rats. Front Pharmacol 2021;12:598959. 10.3389/fphar.2021.598959 33679395 PMC7934060

[64] Xu Y, Li Y, Jadhav K, et al. Hepatocyte ATF3 protects against atherosclerosis by regulating HDL and bile acid metabolism. Nat Metab 2021;3:59–74. 10.1038/s42255-020-00331-1 33462514 PMC7856821

[65] Liu H, Kuang X, Zhang Y, et al. ADORA1 inhibition promotes tumor immune evasion by regulating the ATF3-PD-L1 axis. Cancer Cell 2020;37:324–39. 10.1016/j.ccell.2020.02.006 32183950

[66] Niu F, Yan J, Ma B, et al. Lanthanide-doped nanoparticles conjugated with an anti-CD33 antibody and a p53-activating peptide for acute myeloid leukemia therapy. Biomaterials 2018;167:132–42. 10.1016/j.biomaterials.2018.03.025 29571049 PMC5889738

[67] Almeida AJ, Souto E. Solid lipid nanoparticles as a drug delivery system for peptides and proteins. Adv Drug Deliv Rev 2007;59:478–90. 10.1016/j.addr.2007.04.007 17543416

[68] He W, Yan J, Wang L, et al. A lanthanide-peptide-derived bacterium-like nanotheranostic with high tumor-targeting, -imaging and -killing properties. Biomaterials 2019;206:13–24. 10.1016/j.biomaterials.2019.03.026 30921731 PMC6628696

[69] He W, Yan J, Jiang W, et al. Peptide-induced self-assembly of therapeutics into a well-defined nanoshell with tumor-triggered shape and charge switch. Chem Mater 2018;30:7034–46. 10.1021/acs.chemmater.8b02572 32982042 PMC7518337

[70] He W, Wang S, Yan J, et al. Self-Assembly of therapeutic peptide into Stimuli-Responsive clustered Nanohybrids for Cancer-Targeted therapy. Adv Funct Mater 2019;29:1807736. 10.1002/adfm.201807736 32982625 PMC7518326

[71] Yan J, Yan S, Hou P, et al. A hierarchical peptide-lanthanide framework to accurately redress intracellular carcinogenic protein-protein interaction. Nano Lett 2019;19:7918–26. 10.1021/acs.nanolett.9b03028 31645103

[72] Ghosh P, Han G, De M, et al. Gold nanoparticles in delivery applications. Adv Drug Deliv Rev 2008;60:1307–15. 10.1016/j.addr.2008.03.016 18555555

[73] Dreaden EC, Alkilany AM, Huang X, et al. The golden age: gold nanoparticles for biomedicine. Chem Soc Rev 2012;41:2740–79. 10.1039/C1CS15237H 22109657 PMC5876014

