# Supplementary material for: Nanoparticle delivery of miR-21-3p sensitizes melanoma to anti-PD-1 immunotherapy by promoting ferroptosis
Source: J Immunother Cancer. 2022 Jun 22;10(6):e004381. doi: 10.1136/jitc-2021-004381 (PMC9226924; doi:10.1136/jitc-2021-004381)
Supplement: Supplementary data [file jitc-2021-004381supp001.pdf]

Supplementary Data

Supplementary Materials and Methods

Supplementary Figure Legends

Supplementary Figure S1-S4

Supplementary Materials and Methods

Cell viability analysis

Cell viability was examined by the cell counting kit-8 (CCK-8) assay (7seabiotech, Shanghai, China) following the manufacturer's instructions. In brief, cells were seeded in 96-well plates (5000 cells per well) and received indicated stimulations. The CCK-8 reagent was diluted 1:10 in 100 µl fresh medium for each well and replaced cultured medium after treatment, and then returned to the cell culture incubator for 1 h. The absorbance at 450 nm was measured using a Model 680 Microplate Reader (Bio-Rad, Hercules, CA, USA).

Immunoblotting analysis

After indicated treatment, cells were washed with phosphate-buffered saline (PBS) and lysed with cell lysis solution (RIPA, Beyotime, Shanghai, China), and then total protein extracts were obtained from cells and quantified using BCA Protein Assay kit (Beyotime, Shanghai, China). Equal amounts of protein were separated by SDS polyacrylamide gel electrophoresis (PAGE) and were then transferred onto polyvinylidene fluoride membranes (Millipore, Billerica, MA). After blocking in a solution of 5% non-fat dry milk diluted in Tris-buffered saline, the membranes were washed and then incubated with primary antibodies (cat. 11117-1-AP, rabbit polyclonal anti-TXNRD1, 1:1000, Proteintech, Rosemont, USA; cat.11335-1-AP, rabbit polyclonal anti-IRF1, 1:1000, Proteintech, Rosemont, USA; cat.7649, rabbit monoclonal anti-Phospho-Stat1 (Tyr701) (D4A7) ,1:1000, Cell Signaling Technology, Danvers, MA, USA; cat. ab254268, rabbit monoclonal anti-ATF3, 1:1000, Abcam, Cambridge, MA, USA; cat. 60004-1-AP, mouse monoclonal anti-GAPDH, 1:5000, Proteintech, Rosemont,

USA) overnight at 4°C. After being washed, the membranes were incubated with horseradish peroxidase-conjugated secondary antibodies (Goat Anti-Mouse IgG, 1:5000; Goat Anti-Rabbit IgG, 1:5000, Santa Cruz, CA, USA) for 2 h at room temperature. Bound antibodies were detected using the ECL western blotting detection system and Image J software (Bio-Rad, Hercules, CA, USA).

SiRNA transfection and plasmid vectors

For the transient knockdown of ATF3, siRNAs against *ATF3* were used to transfect melanoma cells. The siRNAs were purchased from GenePharma, Shanghai. The sequences of siRNAs against *ATF3* are as follows: si-ATF3-1, sense 5'-GAGGCGACGAGAAAGAAAUTT-3', antisense 5'-AUUUCUUUCUCGUCGCCUCTT-3'; si-ATF3-2, sense 5'-GCCGAAACAAGAAGAAGGATT-3', antisense 5'-UCCUUCUUCUUGUUUCGGCTT-3'. siRNA transfections were performed using the Invitrogen Lipofectamine siRNAmax Kit according to the manufacturer's instructions. To obtain the stable overexpression of miR-21-3p in B16F10 melanoma cells, the plasmid vectors encoding miR-21-3p were used to transfect cells according to the manufacturer's recommended procedures of Lipofectamine 3000 (Invitrogen). The plasmid vectors were purchased from Genechem, China.

Tumor tissue microarray

Melanoma tissue microarray (DC-Mel21020) was purchased from Avilabio Inc. (Xi'an, China). Detailed information can be accessed via

http://www.avilabio.com/public/details?productId=59764&searchText=DC-Mel21020.

Malondialdehyde (MDA) detection

The MDA level in cell lysates was assessed using a lipid peroxidation assay kit (ab118970, Abcam, Cambridge, MA, USA) according to the manufacturer's instructions. After treatment, cells were washed with ice-cold PBS and homogenized on ice in 300 µl of the MDA lysis buffer. After being centrifuged at 13,000 g for 10 mins, 200 µl of the supernatant from each homogenized sample was transferred to a microcentrifuge tube. Then, 600 µl of the thiobarbituric acid (TBA) solution was added into each vial and incubated at 95°C for 1 h. The MDA in the sample would react with TBA to generate an MDA-TBA adduct. After the samples were cooled to room temperature in an ice bath for 10 mins, 200 µl from each reaction mixture was added into a 96-well plate for analysis. The absorbance was then measured at 532 nm using a Model 680 Microplate Reader (Bio-Rad, Hercules, CA, USA).

Lipid ROS analysis

Lipid ROS level of cells was examined using flow cytometry. In brief, cells were seeded in 6-well plates (3×10^5 per well). After indicated treatment, the culture medium was discarded, and replaced with 5 µM of BODIPY 581/591 C11 (D3861, Invitrogen, Carlsbad, CA, USA) diluted in 1 mL fresh medium for each well, and then incubated at 37 °C for 30 mins. Cells were directly collected and washed with PBS, and then resuspended in 500 µl of PBS. Oxidation of BODIPY-C11 resulted in a shift of the fluorescence emission peak from 590 nm to 510 nm proportional to lipid ROS generation. The fluorescence intensities were quantified

by flow cytometry (BD LSRFortessa™ Flow Cytometer, BD Biosciences, San Jose, CA, USA). A minimum of 10,000 cells was analyzed per condition.

RNA isolation and qRT-PCR

Total RNA was extracted using TRIzol reagent (cat. 15596018, Invitrogen, Carlsbad, CA, USA). mRNA was reversely transcribed to cDNA using the PrimeScript^{TM} RT Master Mix kit (cat. RR036A, TaKaRa, Tokyo, Japan) and qRT-PCR was performed using SYBR Premix Ex TaqTM II kit (cat. RR820A, TaKaRa, Tokyo, Japan). qRT-PCR was performed with a BIO-RAD Multicolor Real-time PCR Detection System (iQTM5, Bio-Rad, Hercules, CA, USA). The primer sequences used for PCR were: h-TXNRD1 forward, 5′-GAGAAAGCTGTGGAGAAGTTTG -3′ and h-TXNRD1 reverse, 5′-CCACAACACGTTCATTGTCTTT -3′; m-TXNRD1 forward, 5’-GTGGCGACTTGGCTAATC-3’ and m-TXNRD1 reverse, 5’-ACCAGGAGAGACACTCAC-3’;ATF3 forward, 5′- TAGCCCCTGAAGAAGATGAAAG -3′ and ATF3 reverse, 5′- CTTCTTCTTGTTTCGGCACTTT -3′; β-actin forward, 5′-TCATGAAGTGTGACGTGGACATC -3′ and β-actin reverse, 5′-CAGGAGGAGCAATGATCTTGATCT -3′. m-GAPDH forward, 5’-TCATCCCAGAGCTGAACG-3’ and m-GAPDH reverse, 5’-TCATACTTGGCAGGTTTCTCC-3’. Relative quantification was performed according to the ΔΔCT method, and results were expressed in the linear form using the formula $2^{-\Delta\Delta CT}$. β-actin mRNA was used as an internal control for human cells. GAPDH mRNA was used as an internal control for murine cells.

Colony formation assay

For colony formation assay, cells were collected, counted, and replated in appropriate dilutions in six-well plate after treatment. After 10-14 days of incubation, colonies were washed with PBS, fixed with 4% paraformaldehyde for 10-20 mins, and then stained with a mixture of 6 % glutaraldehyde and 0.5% crystal violet for 1-2 h. After removing the glutaraldehyde crystal violet mixture carefully and washing with PBS, the plates with colonies were leaved to dry naturally at room temperature. The number of clones was calculated and plating efficiency was determined for each sample.

Transwell invasion and migration assay

Melanoma cells with indicated transfection were incubated at 37 °C for 36 hours. Then, cells were starved in serum-free medium for 12 additional hours and then trypsinized for reseeding on the top chambers of 24-well transwell culture inserts (Corning). After 24 hours, cells were fixed in 4% paraformaldehyde at room temperature for 10 mins. For invasion assays, transwell chambers with 8 μm-pore size membrane filter inserts (Corning) coated with Matrigel (BD Biosciences) were used to determine cell invasion. The non-motile or non-invasive cells on the upper side of the filter were removed, while the motile or invasive cells on the lower side were stained with crystal violet. For the quantification of the invasive and migratory cells, we used the “Multi-point” tool in ImageJ software to count the stained cells. Five fields for each well were counted under the inverted system microscope (Ti-S, Nikon).

Chromatin immunoprecipitation (ChIP) assay

ChIP assay was performed using SimpleChIP® Plus Sonication Chromatin IP Kit (#56383, Cell Signaling Technology, Danvers, MA, USA) according to the manufacturer's instruction. In brief, immunoprecipitation was performed with rabbit monoclonal anti- ATF3 (cat. ab254268, Abcam, Cambridge, MA, USA). Rabbit IgG (cat. ab46540, Abcam, Cambridge, MA, USA) was used as an isotype control antibody. The immunoprecipitated DNA was treated with RNase A and proteinase K and purified using phenolchloroform extraction and ethanol precipitation. Input DNA starting from aliquots of cell lysates was purified using phenol-chloroform extraction and ethanol precipitation. The purified DNA and input genomic DNA were analyzed by real time PCR. Primer sequences of miR-21-3p promoter for amplifying purified DNA in this study are as follows: Forward primer: 5'-AGGTGCCTCCCAAGTTTGCTAATG-3', Reverse primer: 5'-AGAAACTGCCCGCCCTCTCTC-3'. % Input was calculated using 100% × $2^{(-\triangle CT)}$, where△CT = Ct (Ip) – Ct (Input).

Iron assay

Intracellular ferrous iron (Fe^{2+}) level was accessed using the iron assay kit (cat. ab83366, Abcam, Cambridge, MA, USA) according to the manufacturer's recommendations. Briefly, cells after indicated treatment were collected and washed twice with ice-cold PBS. Cells were homogenized in 5× volumes of iron assay buffer on ice. The supernatant was collected and iron reducer was added to each sample before mixing, and then processed to incubation for 30 mins at 37 °C. Then, the iron probe was added to each sample before mixing, and then

processed to incubation for 60 mins at 37ºC. The output was measured immediately on a colorimetric microplate reader at optical density of 593 nm (Promega, Madison, WI, USA).

Supplementary Figure Legend

Supplementary Figure S1

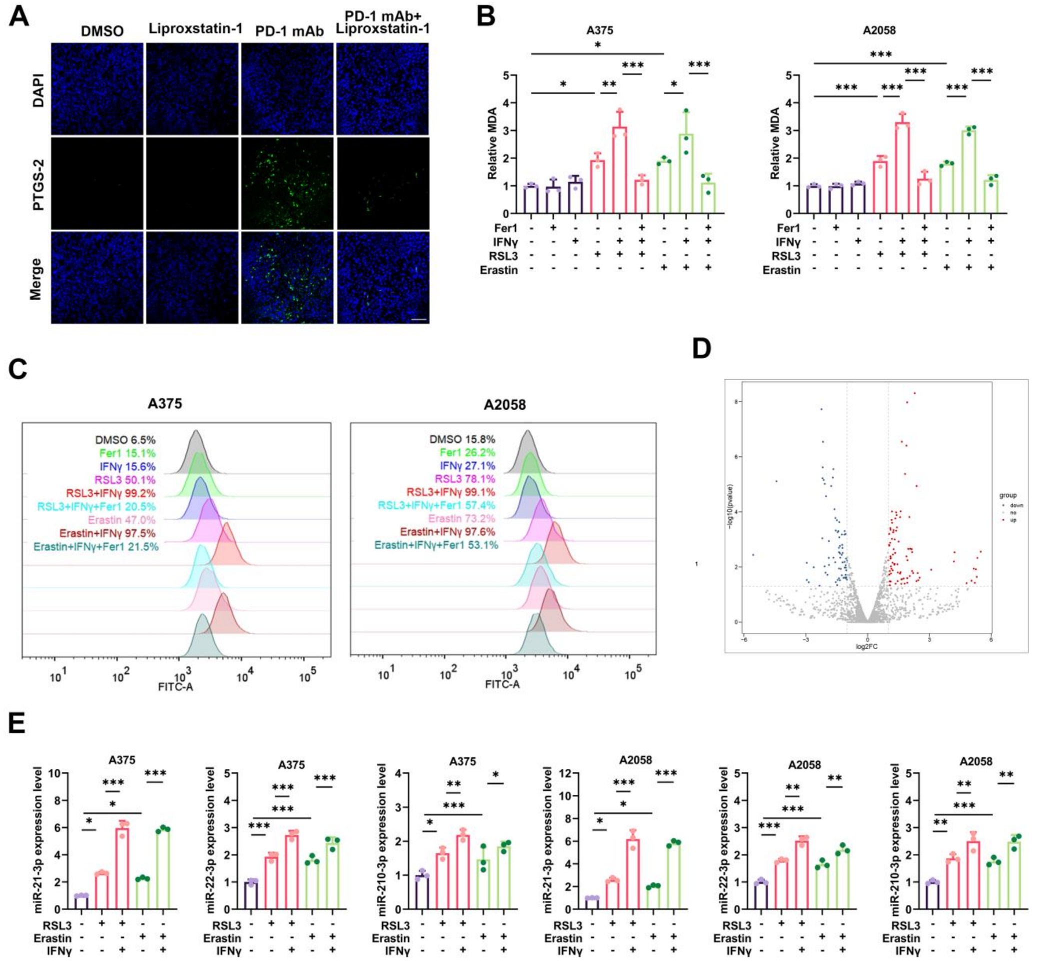

Figure S1. (A) Immunofluorescence staining of PTGS2 in isolated transplanted tumors with indicated treatment. Scale bar = 50μm. **(B)** Relative intracellular MDA content in A2058 and A375 melanoma cells after indicated treatment with IFN-γ, RSL3, Erastin and Fer-1. **(C)** Representative flow cytometry images of relative lipid ROS in A375 and A2058 melanoma cells after indicated treatment with IFN-γ, RSL3, Erastin and Fer-1. **(D)** Volcano of significantly differentially-expressed miRNAs between RSL3-treated group and RSL3+IFN-γ-treated group in A375 melanoma cell through RNA-sequencing. (**E)** The relative expression of the most significantly differentially-expressed miRNAs after the treatment with RSL3/Erastin or combined with IFN-γ in A375 and A2058 melanoma cells.

Erastin was used at 10μM in both cell lines. RSL3 was used at 0.5μM in A375 and 1μM in A2058 cell line. Fer-1 was used at 2μM in both cell lines. IFN-γ was used at 50ng/ml in both cell lines. Data represent the mean ± SD of triplicates. *P* value was calculated by two-tailed Student's t-test. $^{*}P < 0.05$, $^{**}P < 0.01$, $^{***}P < 0.001$. ns, non-significant.

Supplementary Figure S2

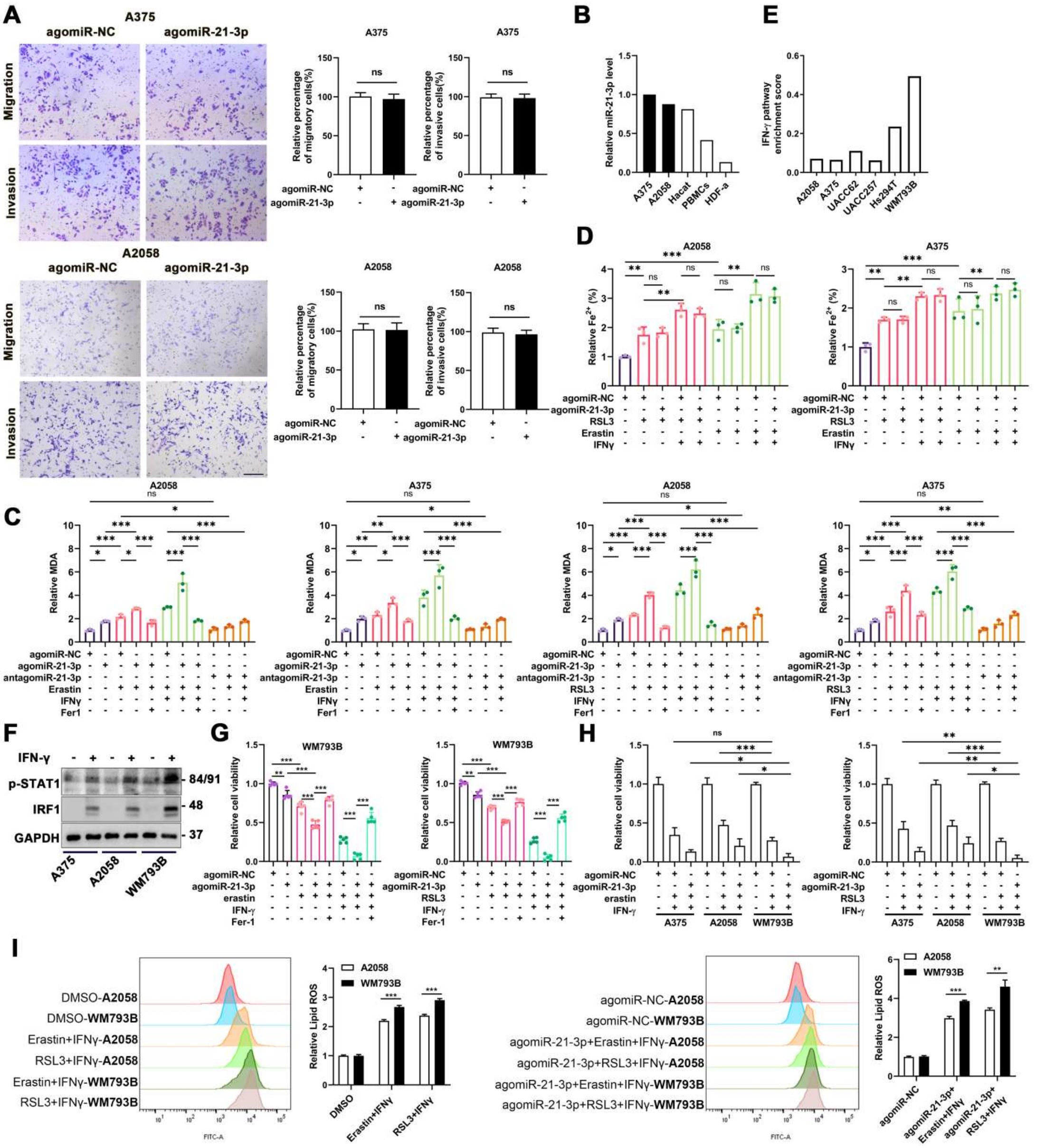

Figure S2. (A) The migration and invasion of A375 and A2058 cells after the treatment with agomiR-21-3p. Scale bar = 100μm. **(B)** Relative level of miR-21-3p in human melanoma cells, keratinocytes, dermal fibroblasts and PBMCs. **(C)** Relative intracellular MDA content in A375 and A2058 melanoma cells after indicated treatment with IFN-γ, RSL3, Erastin and Fer-1. **(D)** Relative Fe^{2+} level in ferroptosis inducer-treated melanoma cells with the intervention of miR-21-3p. **(E)** The enrichment score of IFN-γ pathway in different melanoma cells via GSVA analysis. **(F)** Immunoblotting analysis of p-STAT1 and IRF1 in response to

IFN-γ stimulation (200ng/ml) in three melanoma cell lines. **(G)** Relative cell viability in WM793B melanoma cells after indicated treatment with IFN-γ, RSL3, Erastin and Fer-1. **(H)** The comparison of the relative cell viability between WM793B melanoma cell line and the other two cell lines after indicated treatment. **(I)** The comparison of the relative lipid ROS between WM793B melanoma cell line and the other two cell lines after indicated treatment. AgomiR-NC, agomiR-21-3p and antagomiR-21-3p were all used at 100nM in both cell lines. Erastin was used at 10μM in both cell lines. RSL3 was used at 0.5μM in A375 and 1μM in A2058 cell line. Fer-1 was used at 2μM in both cell lines. IFN-γ was used at 50ng/ml except in the immunoblotting analysis. Data represent the mean ± SD of triplicates. *P* value was calculated by two-tailed Student's t-test. $^{*}P < 0.05$, $^{**}P < 0.01$, $^{***}P < 0.001$. ns, non-significant.

Supplementary Figure S3

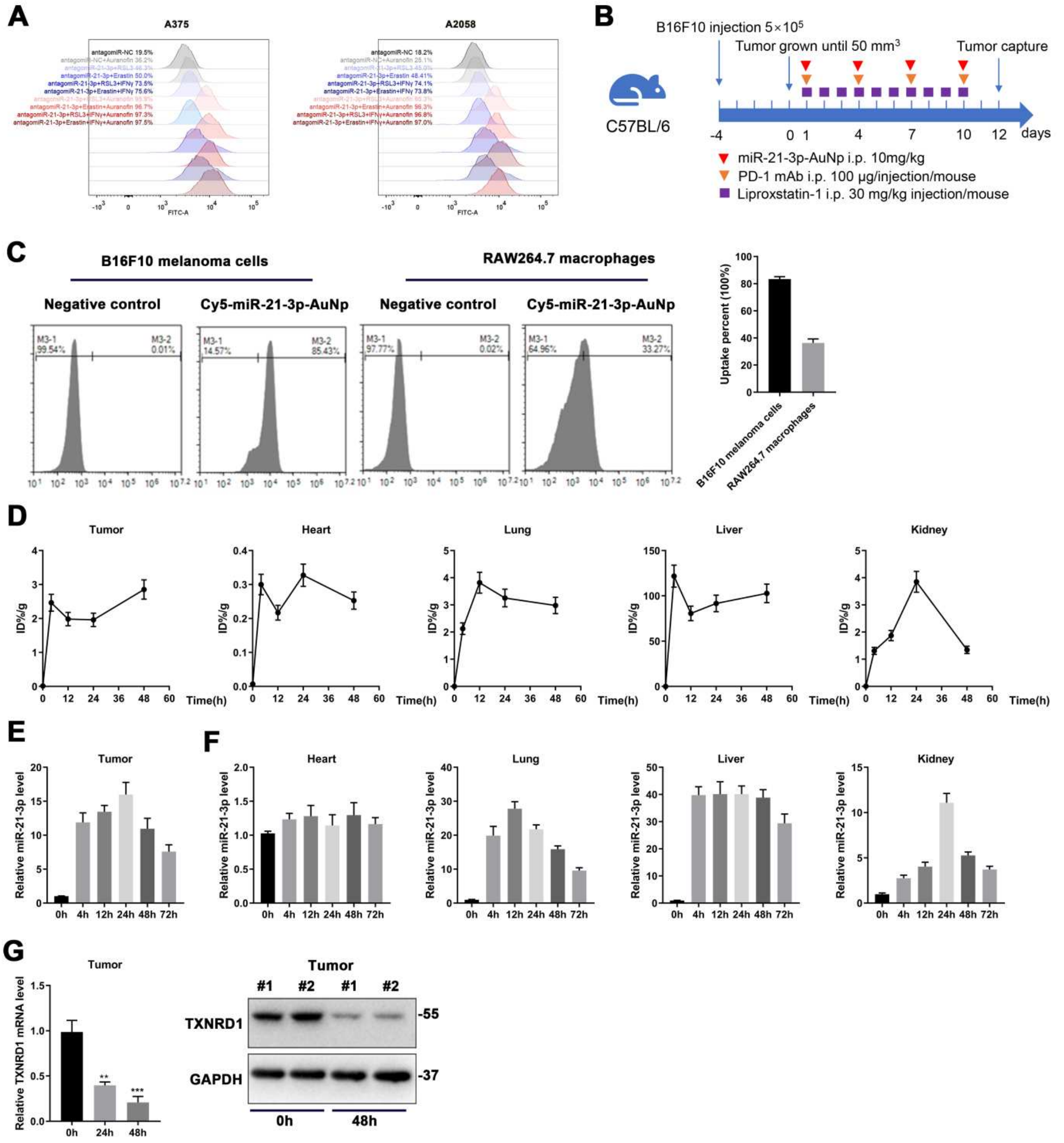

Figure S3. (A) Representative flow cytometry images of relative lipid ROS in A375 and A2058 melanoma cells after indicated treatment related to **Figure 3H**. **(B)** A schematic view of the treatment plan that C57BL/6 mice burdened with B16F10 tumors with or without nanoparticle delivery of miR-21-3p received anti-PD-1 antibody and liprostatin-1 treatment as indicated. **(C)** Flow cytometry analysis and quantification of the uptake of 200nM Cy5-labelled miR-21-3p-AuNp in murine melanoma cells and macrophages after 6 h incubations. **(D)** Tumor and organ distribution of miR-21-3p-AuNp in C57BL/6 mice after

systemic injection of 10mg/kg miR-21-3p-AuNp. Serial sacrifices were carried out at 4h, 12h, 24h and 48h after injection. Several organs/tissues, including heart, liver, lung, kidney and tumor were isolated to determine gold concentrations by ICP-MS. **(E-F)** Relative level of miR-21-3p in tumor and multiple organs after systemic injection of miR-21-3p-AuNp at indicated time points. **(G)** Relative level of TXNRD1 in tumor after 24 hours and 48 hours of the systemic injection of miR-21-3p-AuNp. Immunoblotting detection of TXNRD1 protein level in tumor after 48 hours of the systemic injection of miR-21-3p-AuNp.Two isolated tumors were harvested to perform the analysis. Data represent the mean ± SD of triplicates. *P* value was calculated by two-tailed Student's t-test. $^{*}P < 0.05$, $^{**}P < 0.01$, $^{***}P < 0.001$. ns, non-significant.

Supplementary Figure S4

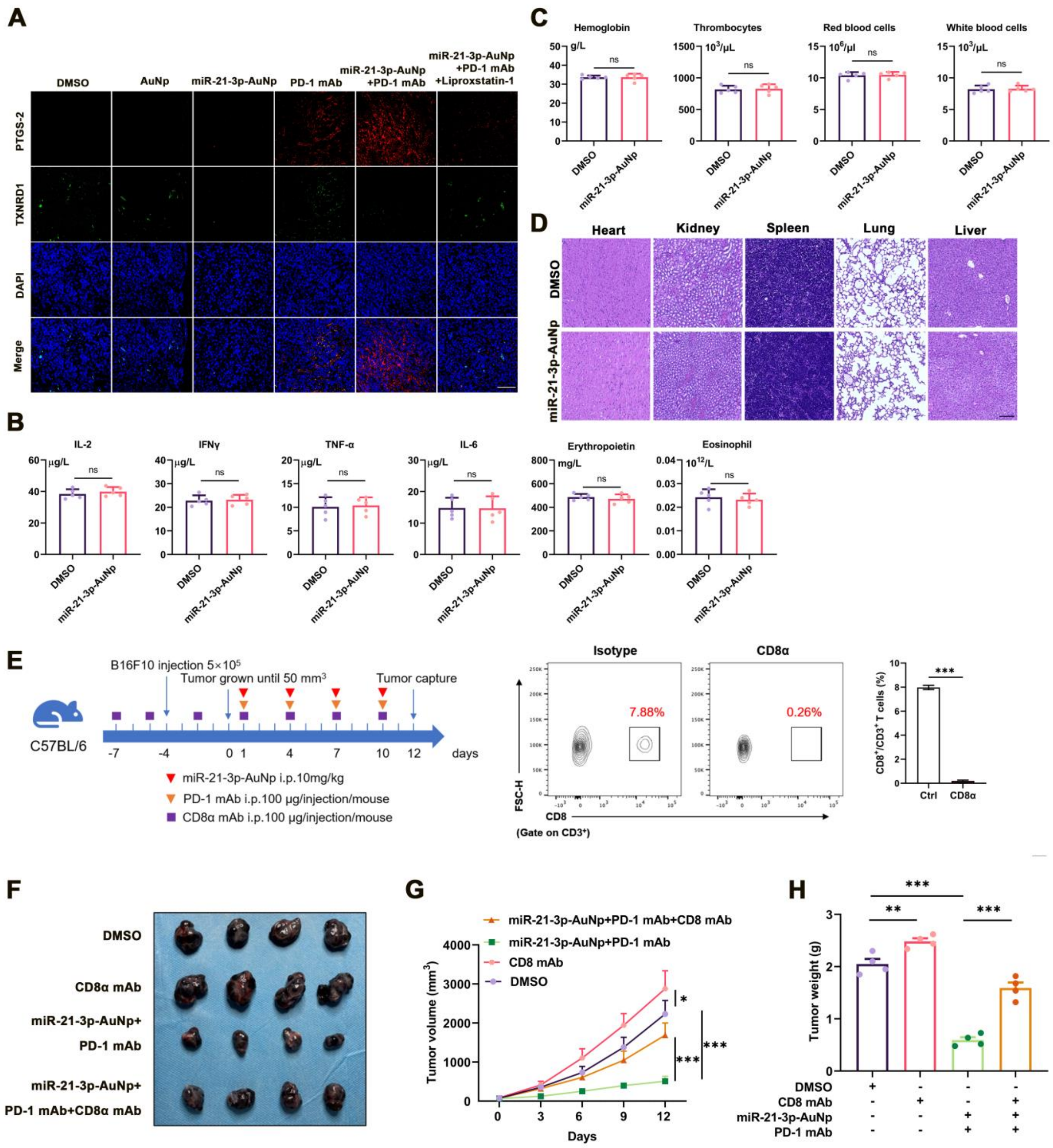

Figure S4. (A) Immunofluorescence staining of PTGS2 and TXNRD1 in isolated transplanted tumors with indicated treatment. Scale bar = 50μm. **(B)** The concentration of IL-2, IFN-γ, TNF-α, IL-6, erythropoietin and eosinophil in serum in C57BL/6 mice receiving miR-21-3p-AuNp or not. **(C)** The absolute content of hemoglobin, thrombocytes, red blood cells and white blood cells in C57BL/6 mice received miR-21-3p-AuNp or not. **(D)** Hematoxylin-eosin staining of heart, kidney, spleen, lung and liver in C57BL/6 mice received miR-21-3p-AuNp or not. Scale bar = 100μm. **(E)** A schematic view of the treatment plan that

C57BL/6 mice burdened with B16F10 tumors with or without nanoparticle delivery of miR-21-3p received anti-PD-1 antibody and CD8α mAb treatment as indicated. **(F-H)** Images of isolated tumors from mice that received indicated treatment. Tumor volumes and weights in each group were calculated and displayed in **(G)** and **(H)**. Data represent the mean ± SD of triplicates. *P* value was calculated by two-tailed Student's t-test. $^{*}P < 0.05$, $^{**}P < 0.01$, $^{***}P < 0.001$. ns, non-significant.
